# Brain Morphometry and Cognitive Performance in Normal Brain Aging: Age- and Sex-Related Structural and Functional Changes

**DOI:** 10.3389/fnagi.2021.713680

**Published:** 2022-01-26

**Authors:** Yauhen Statsenko, Tetiana Habuza, Darya Smetanina, Gillian Lylian Simiyu, Liaisan Uzianbaeva, Klaus Neidl-Van Gorkom, Nazar Zaki, Inna Charykova, Jamal Al Koteesh, Taleb M. Almansoori, Maroua Belghali, Milos Ljubisavljevic

**Affiliations:** ^1^Department of Radiology, College of Medicine and Health Sciences, United Arab Emirates University, Al Ain, United Arab Emirates; ^2^Big Data Analytics Center, United Arab Emirates University, Al Ain, United Arab Emirates; ^3^College of Information Technology, United Arab Emirates University, Al Ain, United Arab Emirates; ^4^Department of Biomedical Engineering, Wayne State University, Detroit, MI, United States; ^5^Department of Obstetrics and Gynecology, Bronxcare Hospital System, Bronx, NY, United States; ^6^Laboratory of Psychology, Republican Scientific-Practical Center of Sports, Minsk, Belarus; ^7^Department of Radiology, Tawam Hospital, Al Ain, United Arab Emirates; ^8^Department of Health and Physical Education, College of Education, United Arab Emirates University, Al Ain, United Arab Emirates; ^9^Department of Physiology, College of Medicine and Health Sciences, United Arab Emirates University, Al Ain, United Arab Emirates

**Keywords:** sex, cognitive decline, aging, executive functioning, psychophysiological test, artificial intelligence, structural-functional association, brain morphometry

## Abstract

**Background:**

The human brain structure undergoes considerable changes throughout life. Cognitive function can be affected either negatively or positively. It is challenging to segregate normal brain aging from the accelerated one.

**Objective:**

To work out a descriptive model of brain structural and functional changes in normal aging.

**Materials and Methods:**

By using voxel-based morphometry and lesion segmentation along with linear statistics and machine learning (ML), we analyzed the structural changes in the major brain compartments and modeled the dynamics of neurofunctional performance throughout life. We studied sex differences in lifelong dynamics of brain volumetric data with Mann-Whitney *U*-test. We tested the hypothesis that performance in some cognitive domains might decline as a linear function of age while other domains might have a non-linear dependence on it. We compared the volumetric changes in the major brain compartments with the dynamics of psychophysiological performance in 4 age groups. Then, we tested linear models of structural and functional decline for significant differences between the slopes in age groups with the *T*-test.

**Results:**

White matter hyperintensities (WMH) are not the major structural determinant of the brain normal aging. They should be viewed as signs of a disease. There is a sex difference in the speed and/or in the onset of the gray matter atrophy. It either starts earlier or goes faster in males. Marked sex difference in the proportion of total cerebrospinal fluid (CSF) and intraventricular CSF (iCSF) justifies that elderly men are more prone to age-related brain atrophy than women of the same age.

**Conclusion:**

The article gives an overview and description of the conceptual structural changes in the brain compartments. The obtained data justify distinct patterns of age-related changes in the cognitive functions. Cross-life slowing of decision-making may follow the linear tendency of enlargement of the interhemispheric fissure because the center of task switching and inhibitory control is allocated within the medial wall of the frontal cortex, and its atrophy accounts for the expansion of the fissure. Free online tool at https://med-predict.com illustrates the tests and study results.

## 1. Introduction

The human brain structure undergoes considerable changes throughout life. Cognitive functioning can also be negatively or positively affected as a consequence of growing and aging. Cognitive tests evidence this by providing a quantitative assessment of the ability to focus (attentional domain), process information, and make a decision (executive functioning, EF, domain).

Researchers aim to expand knowledge on the association of the brain structure with its function at the time of active neurodevelopment, maturation, and decline. It is challenging to work out a descriptive model that segregates normal brain aging from the accelerated one. There are several reasons for this.

First, research on brain aging typically suffers a common limitation: the study cohorts do not cover the whole range of life years (Coffey et al., 1992; Courchesne et al., 2000; Resnick et al., 2000; Preul et al., 2006; Wilke et al., 2007; Aribisala et al., 2013). Some studies dealt with a full age range of selected participants from adolescents, young and midlife adults to the elderly. As an example, one study on the volume of cerebrospinal fluid (CSF) and ventricles examined volunteers from 18 to 80 years. Despite a comprehensive age selection, the study did not have enough subjects of the 5th and 6th decade of life (Gur et al., 1991). Another study included children above 8 years, however, it did not provide any comparison of the significance of age-related changes between different age subgroups (Grieve et al., 2005). A study that combined cross-sectional and longitudinal designs included cohorts similar to ours. But the longitudinal analysis was limited to the age groups of 31–45 years, which might compromise the study outcomes (Narvacan et al., 2017).

Second, individual variability in cognitive performance and neuroplasticity may account for controversial opinions and inconsistency in findings. For instance, prior research indicated that cognitive efficiency depends on the underlying performance ability (i.e., memory speed), former experience with the test, general intelligence (Ram et al., 2005), and personal trail neuroticism (Munoz et al., 2020). Disproportionate exposure to risk factors across the lifespan may translate into a more pronounced risk for cognitive decline (Shaked et al., 2019).

Third, there are many inconsistent findings on the age-associated changes in the major brain compartments and skull volumes, i.e., gray matter (GM), white matter (WM), brain ventricles, and subarachnoid space. As an example, there are contradictory findings on the speed of decline in the GM volume in the older generation (2.37 vs. 0.40 *cm*^3^) (Resnick et al., 2000; Smith et al., 2007). Though it is known that the age-related changes in the WM volume have a U-shape trajectory with almost 10 years of stability, we still do not know whether the decline continues till 50 or 60 years of age as scientists publish conflicting findings (Good et al., 2001; Pagani et al., 2008).

There are no well-established criteria for differentiating between accelerated brain aging and the early stages of neurodegenerative diseases. Neurodegeneration is a common cause of cognitive impairment in older adults. Differentiating and diagnosing these conditions accurately is challenging (Erkkinen et al., 2018). Studies devoted to segregation of Alzheimer's disease (AD) group from the cognitively normal (CN) population show very high accuracy. However, the sensitivity and specificity of the predictive models to segregate AD vs. mild cognitive impairment (MCI) is not that promising (Pellegrini et al., 2018).

### 1.1. Brain Structural Changes in Normal Aging

Both in neuroscience and in medical practice MRI, continues to be the leading image modality for assessing brain structural changes. For this purpose, neuroradiologists employ a set of image sequences, namely T1-weighted (T1W), fluid-attenuated inversion recovery (FLAIR), diffusion-based tractography images (DTI). The brain images acquired with *T1W sequences* can be used for voxel-based brain morphometry (VBM) studies (Liu et al., 2016). *FLAIR sequence* can assess neuroinflammation. *DTI sequence* reflects WM integrity by examining its microstructural components, such as myelination and axonal organization (Asato et al., 2010). Unfortunately, there is a lack of information regarding “normal” WM patterns in children, adolescents, and adults (Weyandt et al., 2013). The issue remains unstudied because of the long time required to obtain DTI data. Authors report low applicability of DTI to studies of small children and the elderly who cannot tolerate it. Moreover, the sequence has a high sensitivity to motion artifacts, this requires sedation in the aforementioned age categories (Hermoye et al., 2006; Dubois et al., 2014).

**Gray matter**. The majority of studies related to reduction in the GM volume concentrated on the regional-specific changes rather than total brain volume (Giorgio et al., 2010; Terribilli et al., 2011; Bourisly et al., 2015). There are controversial data on the accumulation of GM in children and adolescents. While some authors confirmed that the growth happens only until 9 years of age (Courchesne et al., 2000), others found that the GM volume decreases between 9 and 15 years and increases again thereafter (Wilke et al., 2007). A study with both cross-sectional and longitudinal designs presented conflicting data regarding the significance of the association between age and the GM volume (Liu et al., 2003).

**White matter** volume increases until late adulthood and has a certain period of stability which varies in different studies (Courchesne et al., 2000; Pagani et al., 2008). Moreover, studies showed different percentage of decline of WM volume between young and old people (Tang et al., 1997; Liu et al., 2016). White matter hyperintensities (WMHs) are described as lesions that appear as foci or areas of a high intensive signal in MRI FLAIR sequence. As the spread of the lesions across the brain increases throughout life, some authors consider WMHs to be a marker of brain atrophy (Enzinger et al., 2005). However, other researchers argue that WMHs cannot imply a sign of brain aging because the prevalence of the lesions is associated with cerebrovascular diseases which may occur in older people or in young adults (Salat et al., 1999).

**Cerebrospinal fluid** (CSF) cushions the brain and participates in maintaining the homeostasis of the central nervous system. For this reason, CSF circulation is critical for maintaining the brain functions. It also plays a significant role in the functional decline while normal aging (Serot et al., 2003). Some studies reported inconsistent data on the volume of CSF. Such results can not be reliable (Edsbagge et al., 2011). As noted by other investigators, cranial CSF volume in women alters considerably during the menstrual cycle (Grant et al., 1988). Researchers have described an accurate method of measuring total CSF with MRI. The expansion of CSF volume with age provides a good index of brain shrinkage, and the growth of the head confounds the resulting changes.

**Intra- and extraventricular portions of CSF**. Previous reports indicated that brain volume reduces and the volume of the ventricles enlarges with physiological aging, and these findings are substantiated by postmortem data (Gur et al., 1991; Mu et al., 1999). Some studies showed that the values derived from such materials are prone to errors attributable to brain swelling after death or its shrinkage during fixation (Appel and Appel, 1942; Evans, 1942; Dekaban and Sadowsky, 1978). Thus, it is difficult to distinguish true extraventricular CSF from brain. Some attempts have been made to measure the cranial extraventricular CSF volume (Gado et al., 1982; George et al., 1983; Pfefferbaum et al., 1986). It is apparent that the cranial extraventricular CSF space is consistently larger in men (Grant et al., 1991). However, some contradictory evidence from other studies shows no significant association between age and ventricular CSF volume (Mueller et al., 1998; Resnick et al., 2000). Moreover, in crossectional studies, the large amount of between-individual variation in the normal cerebral morphology reduces the sensitivity of methods to detect true cerebral volume differences between groups of subjects of different ages, i.e., brain volumes in distinct sexes (Dekaban and Sadowsky, 1978). Based on other studies, ventricular volume was reported to be larger in older subjects (Scahill et al., 2003). This was also observed in tomographic studies of cerebral atrophy which demonstrated that average ventricular size extends slowly until about the age of 60 years and increases more rapidly thereafter (Zatz et al., 1982; Steiner et al., 1985).

### 1.2. Cognitive Changes in Normal Aging

Age-related decline in cognition are the central topic of studies on neurodevelopment and aging. Much research has been done on this topic. Unfortunately, neuroscience lacks a precise descriptive model of brain structural and functional changes in normal aging.

We performed research to describe the normal dynamics of this central process of brain functioning. Providing such a description will meet the demand of contemporary neuroscience and medicine to give quantitative criteria for these changes. The criteria are expected to be useful for diagnostic purposes. They may facilitate the decision for the physician taking care of patients with early cognitive impairment.

**The application of psychophysiological methods for studying cognition**. Recently, most specialists were skeptical about the effectiveness of such an approach to the early stages of dementia and neurodegenerative disease. This was mainly because of the impossibility of justifying a cut-off value for the majority of cognitive tests. Reasonably, the clinicians consider the tests suitable only for scientific purposes. However, the situation has changed within the last decade because of the huge impact of artificial intelligence on medicine (Habuza et al., 2021a). It provides much promise for utilization of computer-assisted techniques in general medicine and cognitive studies specifically. By conducting research at the populational level, we can observe the tendencies that take place in the society and accumulate the data to be used for machine learning (ML) in diagnostic purposes. It is crucial to work out a set of diagnostic tests that can potentially serve the aforementioned clinical purposes. Applying the results of the tests, one can build up a mathematical model of brain aging in healthy people. The only limitation of such an approach is that it is hard to exclude the early stages of neurodegeneration in the examinees with non-invasive methods. The way to overcome the issue is to use the recent practice parameters of the American Academy of Neurology that recommends resorting to neuroimaging (e.g., MRI) to detect reversible and potentially treatable neurodegenerative conditions (Fuller et al., 2019). The very low number of cases that remain undiagnosed with the radiological studies do not compromise the obtained classification model.

**The methods of studying cognition**. Though there is a diversity of methods and approaches to studying cognition, none of them has been validated as a golden standard. Those ones that are used in clinics for global cognitive function detection, such as mini-mental state examination (MMSE), do not fit in the longitudinal population-based study. The reason is that a single measurement with the MMSE may not fully detect changes in cognition over time (Kim et al., 2019). Its insensitivity to the cognitive impairment from the subcortical lesions in the right hemisphere and the frontal lobes is a considerable limitation to dementia screening. However, some researchers tried to improve its usability by applying ML to the results of a dementia screening questionnaire (Youn et al., 2018). In this study, ML allowed the authors to predict the cognitive impairment with accuracy.

Psychophysiologists use executive functioning tests (EFTs) to estimate cognition, but these methods do not segregate the normal cases from the pathology. Though the idea of EF is stated clearly enough, no concept covers the clinical utility of EF tests (Salthouse et al., 2003; Salthouse, 2005). Researchers have accumulated lots of empirical data on EF changes, but there is no underlying theory that would enrich clinical medicine with a considerable outcome. As a result, now, executive functioning tests (EFTs) are regularly done in medicine exceptionally for scientific purposes.

**Using EF tests in studies on cognition**. The issue of using EFT for estimating cognition was investigated from different perspectives. Studies showed a decrease in functioning in different domains: an overall slowing because of the retardation of information processing, an impairment of working memory, problems with following the concept idea of a discussion because of difficulties in inhibiting irrelevant ideas (task switching and inhibition subdomain), and reduced multitasking abilities (switching subdomain). Additionally, researchers justified the dynamic relationship between physical abilities and cognitive functioning (Clouston et al., 2013). There is an association between sensorimotor variables indicative of biological aging and cognition (Anstey et al., 1997).

Though researchers were looking for a tool to predict an individual's *biological* age and identify adults at increased risk for cognitive impairment, the individual rate of such cognitive changes makes it difficult to establish the associations between age and test performance (Karlamangla et al., 2014). With these tasks, it is challenging to clarify to which extent pathologies account for changes in cognitive subdomains. These tests also do not help to segregate the age- vs. disease-related conditions. For this reason, the need of diagnosing the early stages of neurodegeneration with the help of EFTs remains unmet.

After the analysis of a composite dataset of EFTs of more than 7,000 healthy adults between 18 and 95 years, researchers raised questions about the extent to which a psychophysiological test (PT) measures a distinct dimension of variation in normal adults. After the relation of age to cognitive abilities was taken into consideration, very few EF variables showed any unique relation to age (Salthouse, 2005).

Thus, neurophysiology requires better-designed EFTs and their combinations with other types of examination (e.g., neuroimaging). These combinations will allow us to solve the tasks and specify the diagnosis. One of the possible ways to overcome the limitations of EFTs is to replace the examination with psychophysiological tests (PTs) (Statsenko et al., 2021b). In comparison to EFTs, PTs also cover different cognitive subdomains but do not target any EF specifically. In this way one can analyze which PT reflects this or that cognitive function. But the main idea of PTs is to provide a sensitive tool for qualitative assessment of a functional system (Red'ko et al., 2004; Vityaev and Demin, 2018).

## 2. Objectives

To work out a precise descriptive model in brain structural and functional changes in normal aging, we address the following sub-objectives:

1. To analyze the structural changes of the major brain compartments and to model the dynamics of the neurofunctional performance in the age groups by utilizing an ML approach.
2. To study sex differences in lifelong changes of the brain volumetric data.
3. To select the mathematical model that optimally fits the trajectory of brain structural and functional changes throughout life.
4. To compare volumetric changes in the major brain compartments with the dynamics of psychophysiological performance in age groups.

We covered the lifelong dynamics of the psychophysiological performance in both sexes in recent studies (Statsenko et al., 2019, 2020, 2021c). In this study, we focus on the aspect of sex difference regarding VBM findings.

## 3. Materials and Methods

For the study, we used data from a publicly available dataset (POBA). We collected the dataset in a cross-sectional study design. It consists of about 100 features reflecting the psychophysiological status of the examinees. Here, we give a short description of the study cohort and the features used in the current study. For a detailed explanation of the tests and lifelong dynamics of the dependent variables, refer to our recent publications (Statsenko et al., 2020, 2021a,c,d; Uzianbaeva et al., 2021; Van Gorkom et al., 2021).

### 3.1. Study Participants

While collecting POBA dataset, we scanned 231 people of different ages (4–84 year) with brain MRI and tested them with PTs. The examinees were the healthy participants and the patients who suffered from periodic headaches and were anxious about having organic brain pathology that was excluded later on. The inclusion criterion was literacy, i.e., only those adults who did at least professional courses after finishing general education were involved. The exclusion criteria were as follows: organic brain pathology, mental disorders, injury to the head, radiological signs of neurodegenerative diseases according to the MRI findings. The examinees were not paid for taking part in the study and for the obtained score. The range of years corresponding to age groups is as follows: [0, 20) for Adolescents, [20, 40) for Young adults, [40, 60) for Midlife adults, 60 years and over - for Older adults.

### 3.2. Brain Morphometry

**Figure 1:**
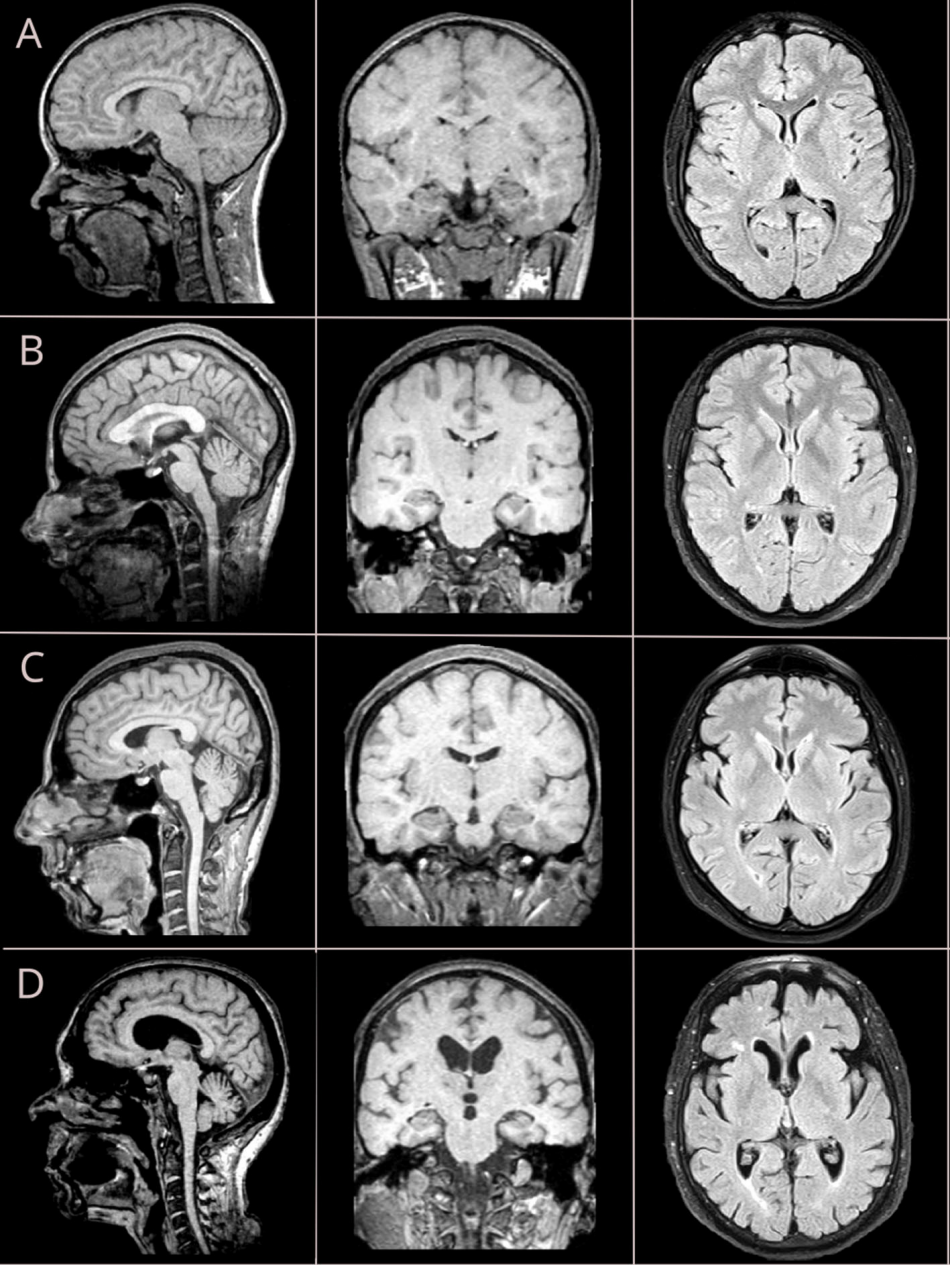
Brain MRI changes across four life periods: adolescent age **(A)**, young adulthood **(B)**, midlife age **(C)**, and older adulthood **(D)**. Sagittal and coronal views are reconstructed from T1-weighted (T1W) sequence with isometric (3D) vowel; axial views are retrieved from fluid-attenuated inversion recovery (FLAIR) sequence.

Brain images were acquired with a 1.5T MRI scanner. For the structural acquisition, we used 3D-T1W images with a voxel size of 1 mm, scanning matrix of 224 × 256, TE of 6.21 ms, TR of 13 ms. For assessing WMHs, we used FLAIR sequence with a slice thickness of 4 mm and scanning matrix of 260 × 320, TE = 104 ms, and TR = 9,130 ms (refer to Figure 1).

To quantify the brain structural changes, we used VBM. To segment the brain into its major compartments (WM and GM, SCF), we resorted to the computational anatomy toolbox (CAT12: http://www.neuro.uni-jena.de/cat/) for the statistical parametric mapping software (SPM: http://www.fil.ion.ucl.ac.uk/spm/). For automated segmentation of changes in FLAIR-hyperintense WM lesions, we used the lesion, segmentation toolbox (LST: https://statistical-modelling.de/lst.html) (Schmidt et al., 2012; Schmidt and Wink, 2017).

### 3.3. Psychophysiological Testing

For testing, we used a battery of neurophysiological tests that reflect the individual psychophysiological status (Statsenko and Charykova, 2010). The battery consisted of a set of tasks involving such cognitive domains as attention and EF, particularly, the subdomain of task switching and inhibitory control. With the tests, one may assess information processing speed. In this study, we modeled changes of the following dependent variables across the lifespan:

1. **Simple visual-motor reaction** (SVMR) is a task with a visual stimulus of a single type and the only way of responding. Mean reaction time (*SVMR_mean*) in a set of subsequent attempts reflects the *information processing speed*.
2. **Complex visual-motor reaction** (CVMR) is a type of the “go/no-go” task, in which the examinee targets one of the two types of triggering stimuli. Mean reaction time (*CVMR_mean*) in this test is normally longer than in SVMR. Decision-making time (DMT) is measured as CVMR_mean subtracted by SVMR_mean (see Equation 1). DMT reflects *task switching and inhibitory control*.
3. **Attention study technique** (AST) is similar to SVMR with an involvement of the *attentional domain*. However, in AST the examinee has to focus on the computer screen because triggering stimuli are presented at different points of time. The tester records average reaction time (*AST_mean*).
4. **Interference resilience technique** (IRT). In the test the examinee is asked to respond to targeting stimuli and skip interfering stimuli that overlap and cover the target. The tester records mean reaction time – *IRT_mean* – in a number of tries. One may calculate *time delay because of visual interference* (TRVI) as Formula 2 states. TRVI reflects *task switching and inhibitory control*.
5. **Reaction to a moving object** (RMO) is a technique assessing the balance between the excitation and inhibition of the central nervous system. Mean reaction time in the test (*RMO_mean*) reflects the predominance of either the excitatory process or the inhibitory process. In the current study, we also analyzed the variance of reaction time across the tries (*RMO_variance*)

(1) $$\begin{array}{l} DMT=CVMR\text{\_}mean-SVMR\text{\_}mean \end{array}$$

(2) $$\begin{array}{l} TRVI=IRT\text{\_}mean-AST\text{\_}mean \end{array}$$

(3) $$\begin{array}{l} CSF\%=CSF/TIV \end{array}$$

(4) $$\begin{array}{l} iCSF\%=iCSF/TIV \end{array}$$

(5) $$\begin{array}{l} GM\%=GM/TIV \end{array}$$

(6) $$\begin{array}{l} WM\%=WM/TIV \end{array}$$

(7) $$\begin{array}{l} WMH\%=WMH/TIV \end{array}$$

### 3.4. Methodology of Study

*To address the first objective*, we studied a pairwise distribution of VBM results (subsection 3.2) across age after adjusting the data to the entire skull volume of the individual as per Formulae 3–7. We built pairwise distributions of psychophysiological attributes (subsection 3.3) across age and analyzed the features related to reaction time, attention, and task switching. For the group analysis, we expressed the results of VBM and PTs for each age cohort and sex as *IQR*, *mean* ± *std*, and compared the distribution of the group data to the entire cohort with the Kruskal-Wallis test.

We studied the relationship of the brain structural features and functional performance with age in consequent life periods. For this, we utilized the Ridge Regression model with a linear least squares optimization function and L2-norm regularization. With a *t*-test, we checked the obtained linear models for significant age-related dynamics (the null-hypothesis stated that a slope value was zero). The dynamics of a variable in an age group were considered significant if the *p*-value for the slope was below the threshold value of 0.05. In the same manner, we tested the slope difference. If *T*-test returned the *p*-value of 0.05 or over, the age-related changes of the variables did not differ significantly in a group. And vice versa, the *p* < 0.05 in the comparison of slopes justified a significant difference in the age-related dynamics of the variables taken into analysis.

*In the second objective*, we assessed the differences between the brain morphometry data for females and males with Mann-Whitney *U*-test. To make the comparison, we also checked if the participants of different sex were distributed equally over age.

*The third objective* was multifold. We inspected associations between the VBM, PTs findings, and age. The hypothesis was that performance in some cognitive domains might decline as a linear function of age while other domains might have a non-linear dependence on age.

*In the first part of objective three*, we explored linear and non-linear relations between the results of VBM, PTs, and age. For this, we composed equations in which a single dependent variable (“feature”) is a function of age:


(8) $$\begin{array}{l} feature=f\left(age\right) \end{array}$$

We used the age exponent as an independent variable:


(9) $$\begin{array}{l} feature=A\cdot age+B \end{array}$$

(10) $$\begin{array}{l} feature=A\cdot age^{2}+B\cdot age+C \end{array}$$

where *A, B, C* ∈ ℝ, and the *feature* can be set to either the structural (e.g., the volume of CSF, GM, WM) or the functional (SVMR_mean, CVMR_mean, DMT, IRT_mean, AST_mean, TRVI, and RMO_mean) attributes.

A regression model presented in Equation (10) is non-linear to age. Despite this, the model remains linear to parameters *A*, *B*, and *C* estimated from the dataset. Hence, for both the linear and non-linear functions (refer to e Equations 9, 10), we could utilize a linear regression algorithm to fit the data. To find parameters for the non-linear function, we generated a new feature matrix that consisted of polynomial features. Then, we fed the linear regression model with the composed matrix. Though this approach allows us to use high dimensional feature spaces, we limited the study to first- and second-order dependencies.

*In the second part of objective three*, we evaluated algorithm performance metrics. This was done to find out which model fits the data best. We used both the linear and the polynomial kernel regression models to convey lifelong changes of VBM and PTs. Both the linear and the parabolic trendline functions were displayed with 95% CIs calculated with the bootstrap method.

Then we compared the performance of the linear and polynomial models to determine which of them generalized the data better. To assess the performance of the models, we used mean absolute error (*MAE*), root mean squared error (*RMSE*), coefficient of determination (*R*^2^) metrics.

*The fourth objective* was to compare the dynamics of the structural and functional changes in the brain in the age groups. Firstly, we analyzed the association between brain volumetric data and cognitive performance. For this purpose, we calculated coefficients of correlation between the data in the entire study cohort. Then, we tested the linear models of brain structural and functional changes for statistically significant differences between the slopes in age groups. To perform the task, we used *t*-test.

### 3.5. Hardware and Software

The calculations were done with the Linux Ubuntu 18.04 workstation with 24 CPU cores and two NVIDIA GeForce GTX 1080 Ti GPU with 11 GB GDDR5X memory each using programming language Python, and its libraries for Data Processing, ML, and Data visualization, such as scikit-learn, NumPy, Pandas, Matplotlib, and Seaborn.

## 4. Results

### 4.1. Brain Structural Changes and Neurofunctional Performance

For detailed analysis of the lifelong dynamics in the skull and brain structure, as well as for the performance in psychophysiological tests refer to Supplementary Material. Brief characteristics of the most remarkable features are below.

#### 4.1.1. Skull and Brain Morphometry Changes Throughout Life

**Figure 2:**
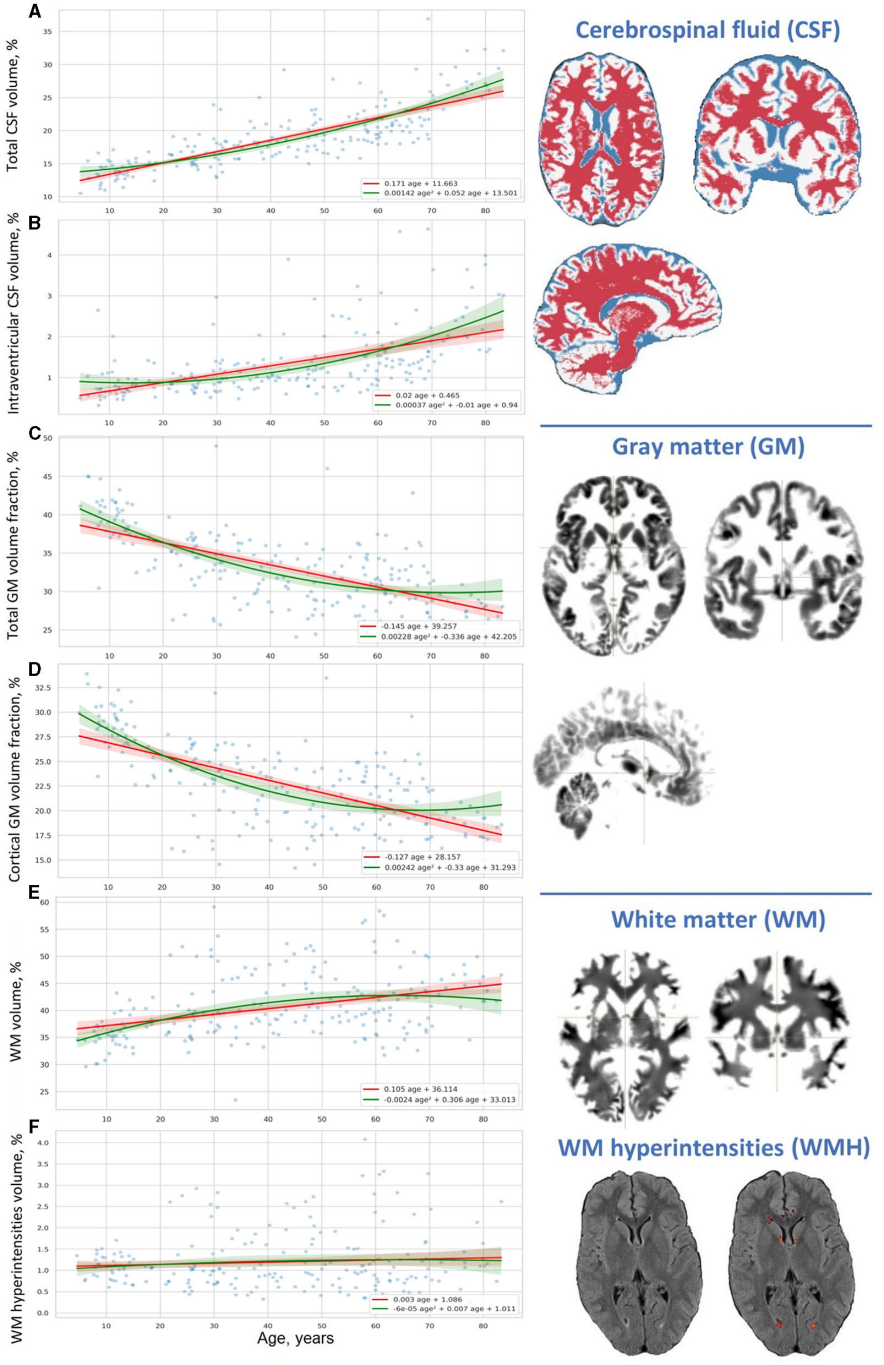
Distribution of results of voxel-based brain morphometry (VBM) over age. **(A)** Total CSF volume, **(B)** intraventricular CSF volume, **(C)** total GM volume fraction, **(D)** cortical GM volume fraction, **(E)** WM volume, and **(F)** WM hyperintensities volume.

**Figure 3:**
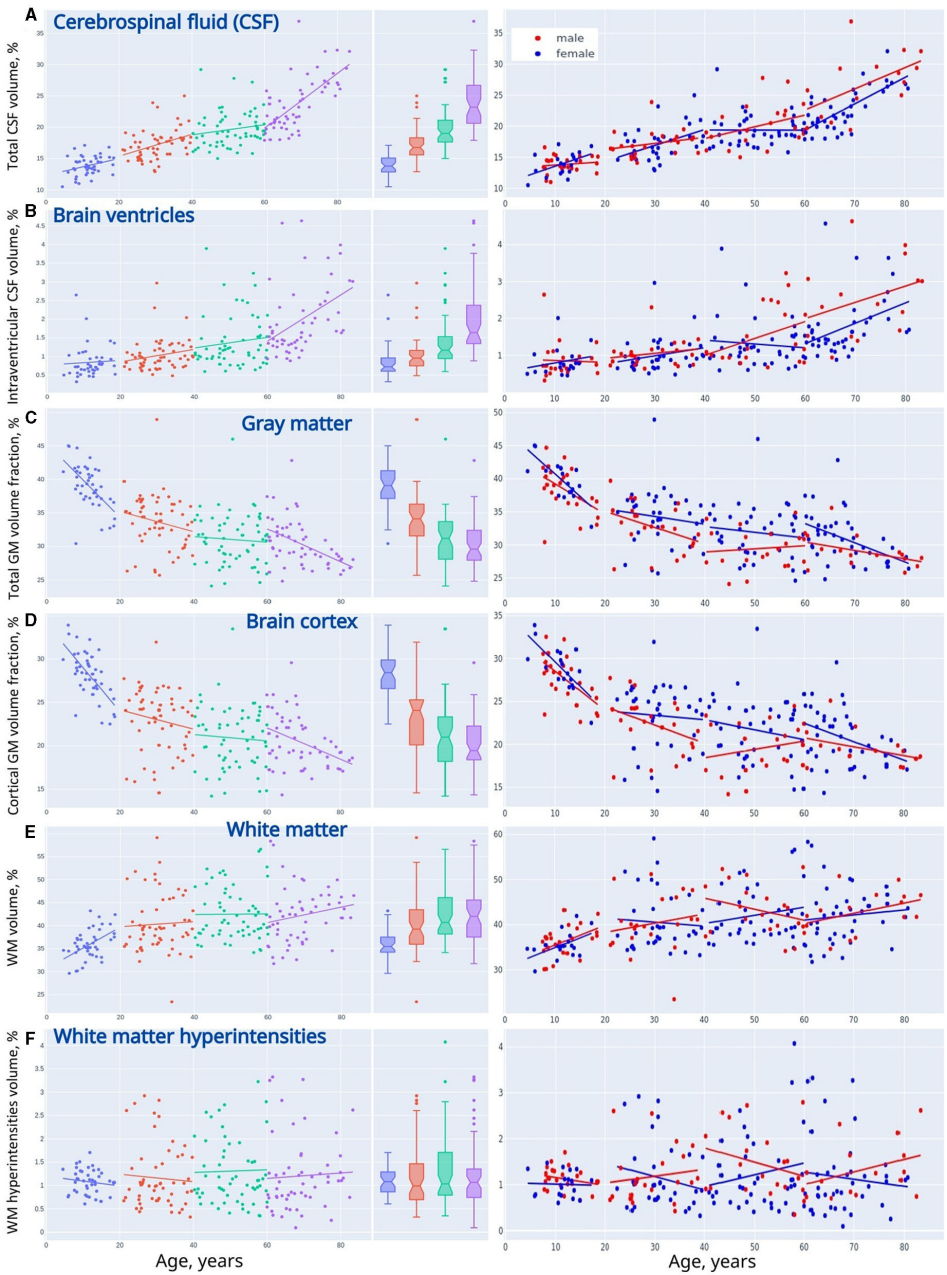
Linear trendlines presenting changes in VBM across life periods (adolescents, young adults, midlife adults, and older adults). **(A)** Cerebrospinal fluid, **(B)** brain ventricles, **(C)** gray matter, **(D)** brain cortex, **(E)** white matter, and **(F)** white matter hyperintensities.

**Figure 4:**
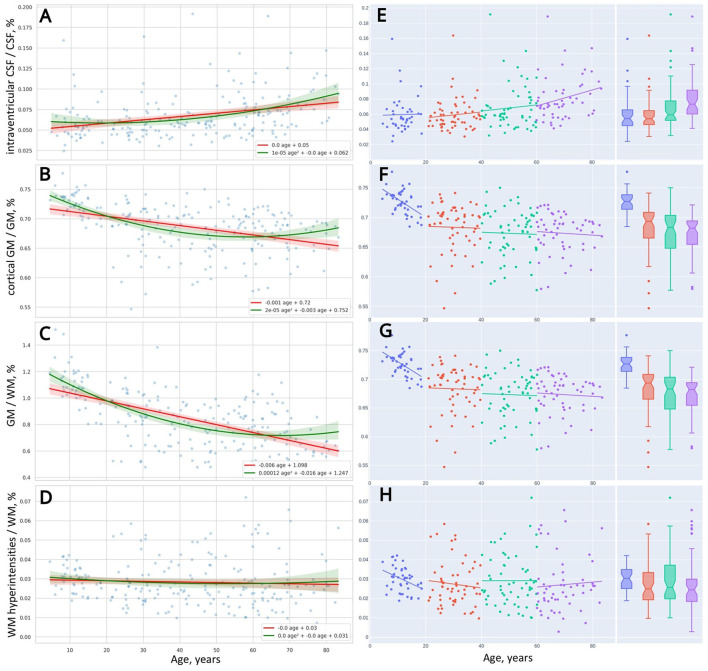
Distribution of brain morphometric indices over age **(A–D)**. Linear trendlines of changes in the indices across four life periods **(E–H)**.

The ordinary least squares (OLS) regression trendlines in Figures 2–4 illustrate a distribution of the major brain compartments over age. The linear regression trendlines in Figures 3, 4E–H show the brain structural changes for each age group. There is a steady decrease in the TIV from Adolescents through Young and Midlife adults to Older adults (*p* = 0.0068). Supposed reasons for this are a rise in the size of the skull and body across generations. The marked difference justifies a necessity to adjust individual brain volume to TIV. This enabled us to perform a comparative study of the age groups.

Table 1 presents the average data for four age groups and the results of Kruskal-Wallis test. The group data with a distribution apparently different from the entire cohort are marked with asterisks. *P*-values in the right column of the table show if there is a distinct difference between all age groups taken into analysis. The bottom part of Table 2 provides a comparison of the slopes for the major brain compartments plotted against age. We inverted the slopes for both GM% and cGM% to compare them with the dynamics of CSF% (refer to data for “*CSF vs*. −GM” and “*CSF vs*. −cGM” in Table 2).

**Table 1:** Skull and brain morphometry in overall study cohort and with regard to age group and sex.

	***All***		***Adolescents***				***Young adults***				***Midlife adults***				***Older adults***				
	**Mean**	**CI**	**mean ±Std**	**Female**	**Male**	**p**	**mean ±Std**	**Female**	**Male**	**p**	**mean ±Std**	**Female**	**Male**	**p**	**mean ±Std**	**Female**	**Male**	**p**	***p*-value**
TIV	1614.42	[1515.99–1724.63]	1653.39 ± 151.51	1555.01 ± 130.92	1724.22 ± 123.11	**0.0002**	1649.87 ± 211.35*	1561.71 ± 192.26	1784.03 ± 162.99	**<0.001**	1597.45 ± 160.86	1528.18 ± 131.34	1722.78 ± 130.75	**<0.001**	1564.78 ± 167.75*	1506.75 ± 137.35	1719.54 ± 141.23	**<0.001**	**0.0068**
CSF	304.51	[249.28–343.44]	229.93 ± 36.1*	217.33 ± 39.12	239.0 ± 30.71	**0.0298**	282.31 ± 52.47*	266.68 ± 48.91	306.08 ± 48.64	**0.0012**	314.81 ± 60.94*	296.61 ± 49.8	347.76 ± 65.29	**0.0011**	375.2 ± 90.53*	343.44 ± 66.61	459.88 ± 91.43	**0.001**	**0.001**
iCSF	21.54	[12.51–24.56]	14.07 ± 7.59*	12.99 ± 6.41	14.84 ± 8.25	0.178	16.74 ± 6.62*	15.39 ± 5.77	18.78 ± 7.28	**0.0173**	22.4 ± 12.47	20.04 ± 10.44	26.68 ± 14.54	**0.0444**	31.53 ± 17.25*	26.77 ± 13.5	44.2 ± 19.6	**0.0005**	**<0.001**
GM	533.15	[462.62–603.28]	640.98 ± 47.03*	616.3 ± 35.47	658.74 ± 46.31	**0.0012**	551.91 ± 76.92*	529.66 ± 69.12	585.76 ± 75.84	**0.0028**	493.04 ± 60.99*	485.2 ± 61.04	507.24 ± 58.28	0.0806	472.1 ± 48.94*	463.46 ± 47.55	495.12 ± 45.0	**0.0176**	**<0.001**
cGM	366.93	[306.94–426.57]	463.22 ± 37.73*	446.42 ± 28.16	475.31 ± 39.1	**0.0077**	377.75 ± 66.71	362.6 ± 65.1	400.81 ± 62.38	**0.0086**	332.26 ± 56.52*	329.67 ± 56.0	336.93 ± 57.14	0.3432	317.43 ± 43.51*	311.19 ± 44.78	334.06 ± 34.82	**0.0491**	**<0.001**
WM	652.5	[566.76–719.3]	595.06 ± 83.52*	551.51 ± 63.94	626.41 ± 81.89	**0.0022**	662.41 ± 122.08	627.54 ± 107.18	715.46 ± 124.25	**0.0034**	676.91 ± 100.46*	642.04 ± 86.79	740.01 ± 92.41	**0.0001**	660.78 ± 116.99	631.51 ± 111.24	738.82 ± 93.84	**0.0011**	**0.0019**
WMH	19.26	[11.81–23.28]	17.8 ± 4.67	15.6 ± 4.1	19.39 ± 4.41	**0.0047**	19.01 ± 10.69	17.87 ± 11.08	20.74 ± 9.82	**0.0492**	20.82 ± 12.16	18.69 ± 12.07	24.67 ± 11.33	**0.0102**	18.99 ± 12.45	17.45 ± 12.77	23.08 ± 10.49	**0.0138**	0.6672
CSF,%	18.88	[15.54-21.25]	13.87 ± 1.53*	13.91 ± 1.8	13.84 ± 1.3	0.4171	17.15 ± 2.44*	17.15 ± 2.68	17.14 ± 2.04	0.3753	19.65 ± 2.86*	19.37 ± 2.49	20.17 ± 3.38	0.2029	23.8 ± 4.25*	22.68 ± 3.33	26.79 ± 4.94	**0.0036**	**<0.001**
iCSF,%	1.33	[0.79–1.51]	0.84 ± 0.42*	0.83 ± 0.38	0.85 ± 0.45	0.4559	1.02 ± 0.42*	1.01 ± 0.46	1.04 ± 0.35	0.1508	1.38 ± 0.7	1.3 ± 0.63	1.53 ± 0.77	0.2406	1.97 ± 0.93*	1.75 ± 0.79	2.54 ± 1.02	**0.0033**	**<0.001**
GM,%	33.15	[29.18–36.46]	38.97 ± 3.24*	39.82 ± 2.87	38.36 ± 3.36	*0.0715*	33.66 ± 4.0	34.19 ± 4.25	32.86 ± 3.44	*0.0935*	31.0 ± 3.79*	31.85 ± 3.96	29.46 ± 2.86	**0.009**	30.37 ± 3.48*	30.94 ± 3.65	28.87 ± 2.38	**0.0145**	**<0.001**
cGM,%	22.8	[19.26–25.86]	28.18 ± 2.7*	28.87 ± 2.53	27.68 ± 2.71	0.0981	23.0 ± 3.55	23.35 ± 3.79	22.48 ± 3.09	0.1046	20.89 ± 3.57*	21.64 ± 3.68	19.54 ± 2.91	**0.0142**	20.43 ± 3.03*	20.78 ± 3.26	19.49 ± 2.01	*0.101*	**<0.001**
WM,%	40.52	[35.98–43.85]	35.93 ± 3.18*	35.46 ± 2.85	36.26 ± 3.35	0.1211	40.33 ± 6.28	40.47 ± 6.4	40.12 ± 6.08	0.2573	42.49 ± 5.65*	42.17 ± 5.71	43.08 ± 5.49	0.1985	42.21 ± 5.89*	41.91 ± 6.29	43.01 ± 4.56	0.1847	**<0.001**
WMH,%	1.19	[0.75–1.39]	1.08 ± 0.26	1.01 ± 0.27	1.12 ± 0.24	0.1366	1.16 ± 0.66	1.15 ± 0.71	1.17 ± 0.58	0.2422	1.31 ± 0.78	1.23 ± 0.81	1.45 ± 0.71	*0.0737*	1.2 ± 0.77	1.15 ± 0.82	1.34 ± 0.59	*0.0883*	0.6518
iCSF/CSF	6.73	[4.94–7.53]	5.95 ± 2.39*	5.77 ± 1.87	6.09 ± 2.69	0.3885	5.92 ± 2.07*	5.85 ± 2.39	6.02 ± 1.45	0.1075	6.86 ± 2.87	6.61 ± 2.81	7.32 ± 2.92	0.2659	8.04 ± 2.81*	7.59 ± 2.72	9.25 ± 2.7	**0.0076**	**<0.001**
cGM/GM	68.66	[66.48–71.58]	72.61 ± 1.97*	72.76 ± 1.81	72.51 ± 2.08	0.2415	68.34 ± 4.01	68.26 ± 4.63	68.47 ± 2.82	0.2227	67.31 ± 4.03*	67.84 ± 3.8	66.36 ± 4.27	0.116	67.35 ± 3.32*	67.19 ± 3.63	67.78 ± 2.24	0.4736	**<0.001**
GM/WM	84.61	[66.5–100.54]	109.53 ± 15.05*	113.29 ± 14.76	106.82 ± 14.67	*0.0785*	86.12 ± 19.05	86.91 ± 17.98	84.92 ± 20.52	0.2323	74.84 ± 15.8*	77.43 ± 15.81	70.17 ± 14.65	**0.0375**	74.01 ± 16.8*	76.18 ± 17.77	68.22 ± 12.14	*0.0741*	**<0.001**
WMH/WM	2.83	[2.0–3.41]	2.98 ± 0.64*	2.82 ± 0.65	3.1 ± 0.6	*0.0785*	2.74 ± 1.16	2.7 ± 1.26	2.8 ± 0.98	0.2134	2.92 ± 1.33	2.76 ± 1.37	3.22 ± 1.19	*0.0595*	2.71 ± 1.43	2.58 ± 1.51	3.04 ± 1.1	*0.0511*	*0.0602*

*Structural features are reported in cm^3^ or % of TIV. An asterisk is used to mark variables with distribution significantly different (p < 0.05) in age group compared to overall study cohort*.

*Significant differences between sexes and among age groups (p < 0.05) are marked in bold font*.

**Table 2:** Interaction coefficients for comparison of slopes for psycho-physiological performance and brain morphometry.

	**SLOPE**											
	***Adolescents***			***Young adults***			***Midlife adults***			***Older adults***		
	**Estimate ±Std**	**CI**	***p***	**Estimate ±Std**	**CI**	***p***	**Estimate ±Std**	**CI**	***p***	**Estimate ±Std**	**CI**	***p***
*SVMR_mean*	–14.1 ± 2.51	[–19.2;–9.03]	**1.51e-06**	2.34 ± 0.687	[0.969 ± 3.72]	**0.0012**	0.127 ± 1.18	[–2.25 ± 2.5]	0.915	1.88 ± 1.09	[–0.301;4.06]	0.09
*CVMR_mean*	–20.6 ± 3.96	[–28.6;–12.7]	**5.52e-06**	6.83 ± 1.17	[4.48 ± 09.18]	**2.96e-07**	0.913 ± 1.36	[–1.8 ± 3.63]	0.504	2.53 ± 1.45	[–0.375;5.43]	0.086
*DMT*	–6.542 ± 2.264	[–11.1–1.97]	**0.00615**	4.49 ± 1.11	[2.26 ± 6.72]	**0.0001705**	0.786 ± 1.15	[–1.51;3.08]	0.496	0.702 ± 1.2	[–1.7;3.1]	0.56
*AST_mean*	–12.5 ± 2.3	[–16.8;–8.39]	**2.6e-07**	3.91 ± 1.11	[1.7 ± 6.13]	**0.000818**	1.43 ± 1.34	[–1.24;4.11]	0.288	0.32 ± 01.26	[–2.22;2.86]	0.801
*IRT_mean*	–17.0 ± 3.06	[–23.2;–10.9]	**1.791e-06**	4.52 ± 1.02	[2.48 ± 6.57]	**4.4e-05**	2.41 ± 1.46	[–0.507;5.33]	0.104	3.24 ± 01.49	[0.241;6.24]	**0.035**
*TRVI*	–4.55 ± 2.8	[–10.2;1.11]	0.1118	0.61 ± 1.02	[–1.44 ± 2.66]	0.554	0.98 ± 0.986	[–0.994 ± 2.95]	0.324	2.92 ± 1.49	[–0.065;5.9]	0.055
*RMO_mean*	–6.34± 3.1	[–12.6;–0.073]	**0.0475**	–0.397 ± 1.43	[–3.27 ± 2.48]	0.783	–1.32 ± 2.33	[–5.98;3.34]	0.573	2.45 ± 1.53	[–0.628;5.52]	0.116
*iCSF*	0.006 ± 0.019	[–0.033–0.045]	0.752	0.017 ± 0.011	[–0.004 ± 0.038]	0.11	0.014 ± 0.014	[–0.016 ± 0.045]	0.351	0.062 ± 0.017	[0.027;0.097]	**0.000758**
*CSF*	0.135 ± 0.067	[–0.001-0.270]	0.051	0.175 ± 0.058	[0.058 ± 0.292]	**0.00405**	0.081 ± 0.062	[-0.043;0.205]	0.195	0.438 ± 0.065	[0.308 ± 0.567]	**1.04e-08**
*iCSF / CSF*	0.01009 ± 0.011	[–0.211;0.232]	0.927	0.039 ± 0.053	[–0.067;0.145]	0.466	0.04 ± 0.063	[–0.086;0.165]	0.528	0.105 ± 0.057	[–0.009;0.218]	*0.069*
*WMH*	0.011 ± 0.012	[–0.035;0.013]	0.379	–0.008 ± 0.017	[–0.042 ± 0.026]	0.637	0.03 ± 0.017	[–0.031 ± 0.037]	0.868	0.006 ± 0.016	[–0.026 ± 0.038]	0.72
*WM*	0.448 ± 0.128	[–0.889 ± 0.379]	**0.00115**	–0.02 ± 0.03	[–0.08; 0.04]	0.504	0.0004889 ± 0.029	[–0.058;0.059]	0.987	0.013 ± 0.03	[–0.047;0.072]	0.671
*WMH / WM*	–0.067 ± 0.027	[–0.123;–0.012]	**0.019**	0.055 ± 0.161	[–0.268; 0.379]	0.733	0.006 ± 0.124	[–0.242;0.254]	0.96	0.167 ± 0.12	[–0.074 ±0.409]	0.17
*cGM*	–0.512 ± 0.096	[–0.706; –0.318]	**3.82e-06**	–0.114 ± 0.09	[–0.294 ± 0.067]	0.213	–0.036 ± 0.078	[–0.192 ± 0.12]	0.644	–0.179 ± 0.058	[–0.295 ± –0.064]	**0.00302**
*GM*	–0.552 ± 0.122	[–0.798 ± –0.307]	**4.78e-05**	–0.151 ± 0.101	[–0.353 ± 0.051]	0.14	–0.041 ± 0.083	[–0.207 ± 0.125]	0.622	–0.246 ± 0.064	[–0.374 ± –0.118]	**0.0003165**
*cGM / GM*	–0.306 ± 0077	[–0.462;–0.151]	**0.000276**	–0.017 ± 0.103	[–0.224;0.19]	0.872	–0.018 ± 0.088	[–0.195;0.159]	0.838	–0.03 ± 0.069	[–0.168;0.108]	**0.668**
**SLOPE DIFFERENCE BETWEEN MORPHOLOGICAL BRAIN ESTIMATES**												
*iCSF vs. CSF*	0.128 ± 0.07	[–0.01 ± 0.267]	0.069	0.158 ± 0.059	[0.041 ± 0.276]	**0.00879**	0.067 ± 0.064	[–0.05;0.193]	0.296	0.376 ± 0.067	[0.243 ± 0.508]	**1.57e–07**
*CSF vs. –GM*	0.418 ± 0.139	[0.142 ± 0.694]	**0.00346**	–0.024 ± 0.117	[–0.255 ± 0.207]	0.837	–0.04 ± 0.103	[–0.245;0.165]	0.7	–0.192 ± 0.091	[–0.372 -0.012]	**0.0372**
*CSF vs. GM*	–0.687 ± 0.139	[–0.963 ± –0.411]	**3.9e-06**	–0.326 ± 0.117	[–0.557 ± –0.095 ]	**0.00609**	–0.122 ± 0.103	[–0.327 ± 0.083 ]	0.23995	–0.684 ± 0.091	[–0.864 ± -0.504 ]	**1.79e-11**
*CSF vs. -cGM*	0.377 ± 0.117	[0.144;0.61]	**0.00182**	–0.062 ± 0.107	[–0.274;0.151]	0.567	–0.045 ± 0.1	[–0.242;0.152]	0.654	–0.258 ± 0.087	[–0.43;–0.087]	**0.00354**
*CSF vs. cGM*	–0.647 ± 0.117	[–0.879;–0.414 ]	**3.83e-07**	–0.289 ± 0.107	[–0.502;–0.076 ]	**0.00827**	–0.117 ± 0.1	[–0.314;0.08 ]	0.241	0.617 ± 0.087	[– 0.789;–0.445 ]	**1.32e–10**
*CSF vs. WM*	0.313 ± 0.145	[0.26;0.601]	**0.033**	–0.12 ± 0.172	[–0.46;0.22]	0.487	–0.075 ± 0.138	[–0.349;0.199]	0.590	– 0.27 ± 0.136	[–0.541;0]	**2.2e–16**
*CSF vs. WMH*	–0.145 ± 0.068	[–0.281;–0.011]	**0.036**	–0.183 ± 0.061	[–0.304;–0.063]	**0.003**	–0.078 ± 0.064	[–0.205;0.049]	0.226	–0.432 ± 0.067	[–0.564;–0.3]	**2.2e–16**
*cGM vs. GM*	–0.04 ± 0.155	[–0.349;0.268 ]	0.794	–0.038 ± 0.135	[–0.306;0.231]	0.782	–0.005 ± 0.114	[–0.23;0.22]	0.966	–0.067 ± 0.086	[–0.237;0.104]	0.44
*GM vs. –WM*	–0.105 ± 0.177	[–0.456;0.247]	**2.2e–16**	–0.096 ± 0.19	[–0.473;0.282]	0.616	–0.035 ± 0.149	[–0.33;0.26]	0.816	–0.079 ± 0.136	[–0.348;0.191]	**0.565**
*GM vs. WM*	1 ± 0.177	[0.649;1.35]	**2.12e-07**	0.206 ± 0.19	[–0.171;0.584 ]	0.281	0.047 ± 0.149	[–0.248;0.342 ]	0.751	0.413 ± 0.136	[0.144;0.683 ]	**0.00301**
*WM vs. WMH*	–0.458 ± 0.129	[–0.714;0.202]	**0.000615**	–0.063 ± 0.162	[–0.385;0.258]	0.697	–0.003 ± 0.125	[–0.251;0.244]	0.978	–0.162 ± 0.121	[–0.402;0.079]	0.185

*Structural features are reported in % of TIV. Significant differences between slopes (p < 0.05) are marked in bold font. CI – 95% confidence interval*.

The percentage of the CSF (*CSF%*) increases placidly throughout life and its accumulation reflects the atrophy of the brain parenchyma. For all life periods the speed of enlargement of the intracranial subarachnoid space is higher compared to that of the brain ventricles.

A decrease in the ratio of GM (GM%) to the TIV is observed throughout life. In Adolescents, a reversed slope for the *GM%* loss across the lifespan is pronouncedly steeper than a slope for the *CSF%* accumulation. An accumulation of WM at the time of active neurodevelopment and myelination may compensate for the loss of GM. In Older adults, a reversed slope for the *GM%* loss is significantly shallower than a slope for the *CSF%* accumulation. The supposed explanation is related to the speed of the WM accumulation which is fast in minors and reduced at advanced age. The percentage of cortical gray matter (cGM%) to TIV reduces with advancing age.

The percentage of total WM (*WM%*) follows an age-related pattern of changes opposite to the one for *GM%*. It rises across the lifespan but the rate of changes is not common for the age groups. The expansion of WM is substantial in Adolescents. This is followed by a slight increase in *WM%* from 20 years till the end of life. The polynomial model of the distribution of the WM volume over age evidences a shallow decrease in the volume after the age of 60–65 years.

Vascular lesions of WM are not the major determinant of the life-long structural changes in the brain. They should be considered as a sign of brain disease rather than a common outcome of brain aging. An increase in *CSF%* continues throughout life and has its peak in Older adults, whereas the fraction of the TIV occupied by WMHs (*WMH%*) remains almost unchanged in normal brain aging. In all the age groups, the linear trendlines are shallower for *WMH%* compared to *CSF%*.

When adjusted to the skull volume, the relative volumes of the brain compartments (e.g., *GM%*, *WM%*, *CSF%*, etc.) do not differ evidently for both sexes in Adolescents and Young adults. The tendency changes after 40 years of age. The sex-related differences can be explained by the disparity either in the speed or in the onset of the atrophic changes in GM. It starts earlier or goes faster in males. A marked difference in the proportion of total CSF and iCSF proves that elderly men are prone to age-related brain atrophy.

#### 4.1.2. Performance in Psychophysiological Tests With Age

**Figure 5:**
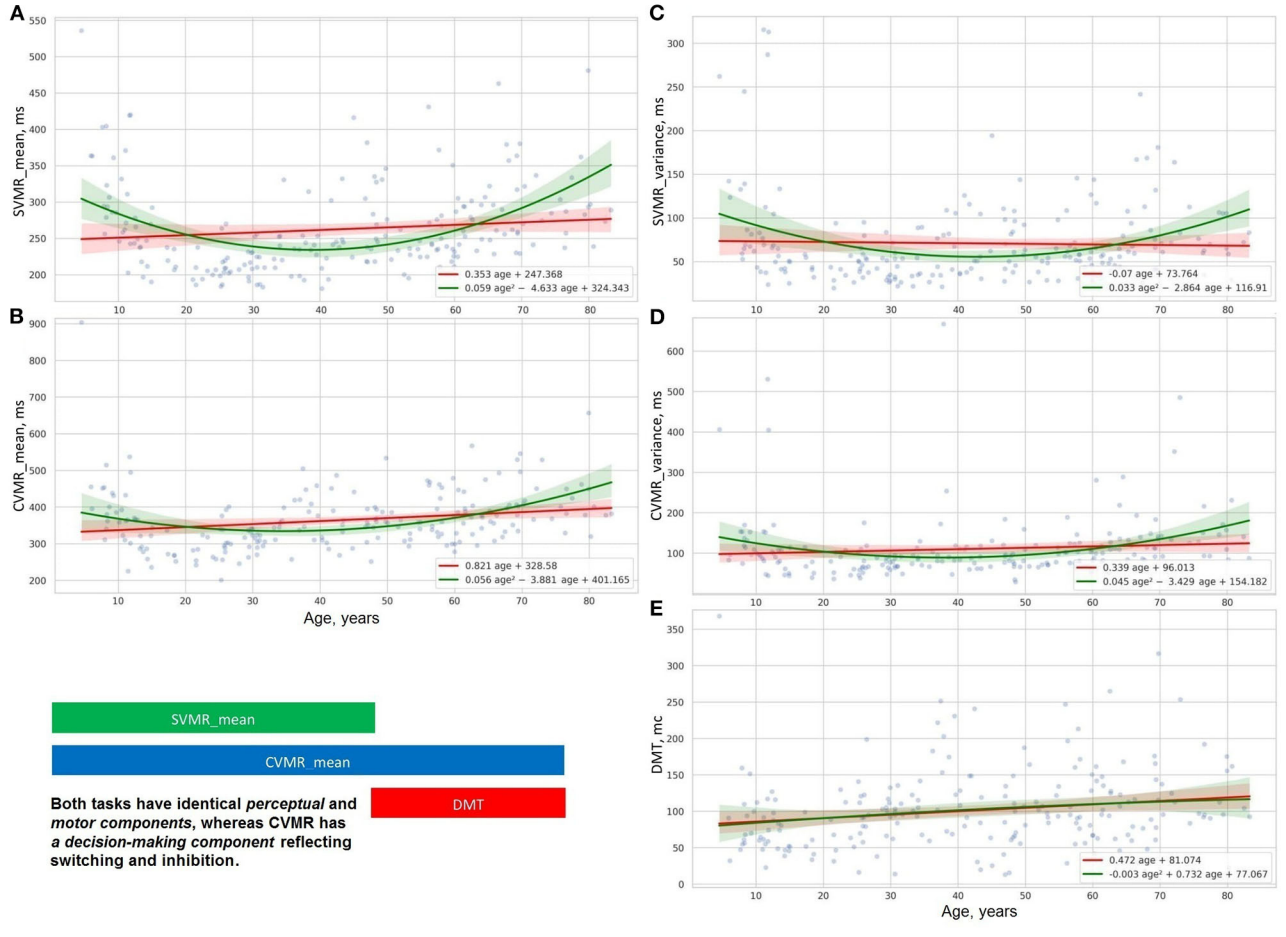
Distribution of decision making **(E)**, reaction time and its varience in simple **(A,C)**, and complex **(B,D)** visual-motor tasks.

**Figure 6:**
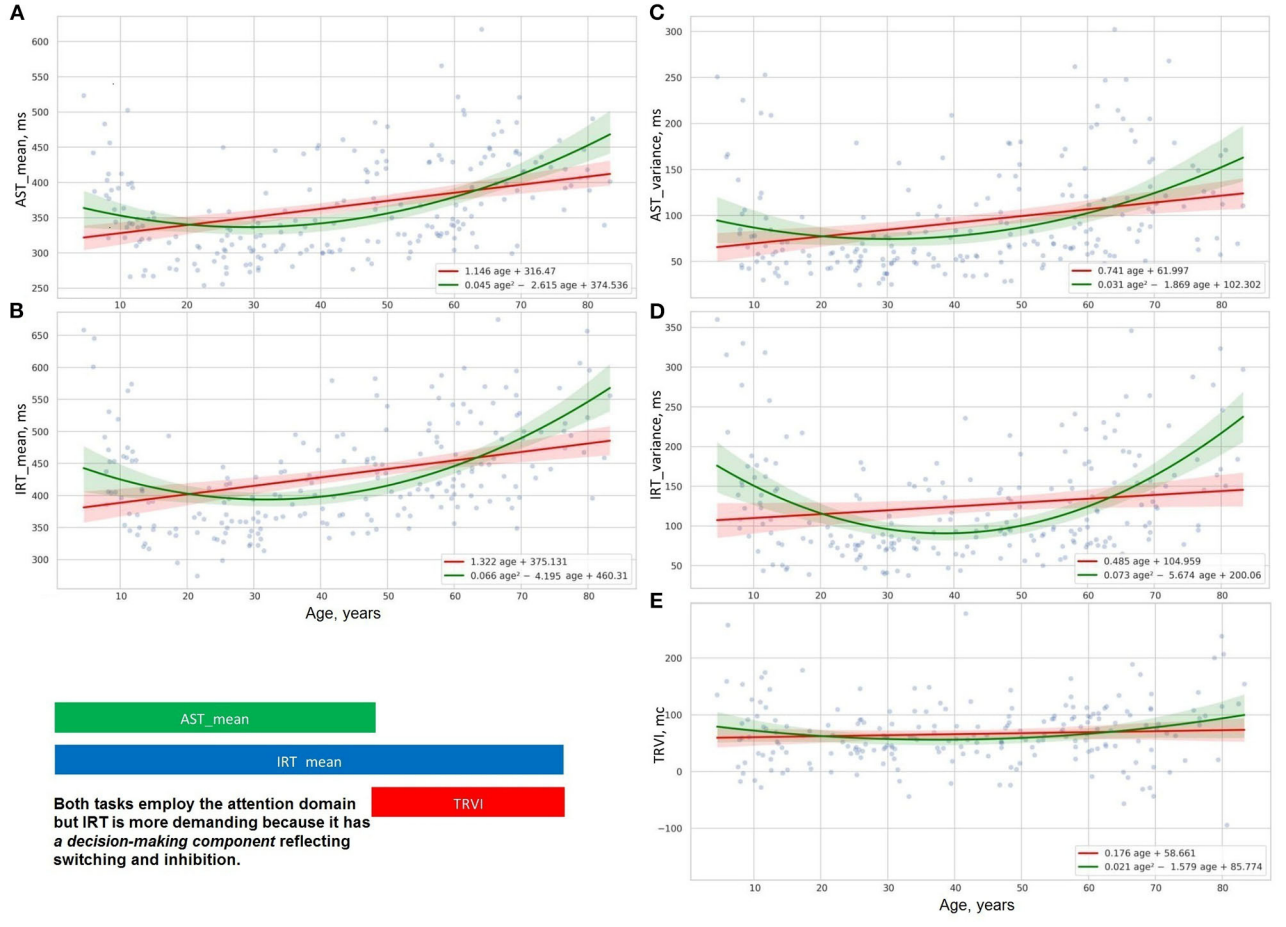
Distribution of time delay because of distraction **(E)**, reaction time and its variance in studies of attention with **(B,D)**, and without interference **(A,C)**.

**Figure 7:**
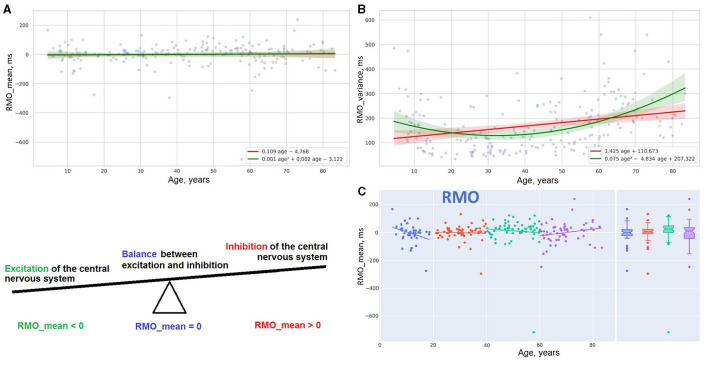
Distribution of reaction time and its variance in RMO task **(A,B)**. Linear trendlines of performance in RMO task in age groups **(C)**.

The distribution of psychophysiological tests results over age is illustrated with OLS regression trendlines in Figures 5–7. The top rows of Table 2 shows a comparison of the slopes for the PTs results plotted against age.

Figures 5A–D, 6A–D show the age-related changes of the dependent variables that reflect information processing speed in PTs with diverse task paradigms. The distribution of the test results over age fits better the parabolic trendline colored in green than the linear trendline drawn in red.

Figures 5E, 6E present the distribution of the derivative variables that reflect the time spent on task switching and inhibitory control (i.e., inhibiting an automatic response, making a decision, selecting the correct way to respond, etc.). The data on these scatterplots have almost linear distribution that is close to the red linear trendline.

The data obtained justify the presence of distinct patterns of age-related changes in cognitive functions. A supposed reason for this is that cognitive domains do not have a common structural representation in the brain and the structural correlates undergo disparate age-related changes. Most studies concentrate on the rate of progression or reduction in cognitive performance, whereas the pattern of the age-related changes is specific to the cognitive domain and should be also considered.

The results of the psychophysiological tests that reflect information processing speed follow a “U-shaped” pattern of changes. The age-related variability of reaction time in SVMR, CVMR, AST, and IRT tests is properly explained with polynomial kernel ML models.

Psychophysiological metrics reflective of task switching and inhibitory control (e.g., DMT, the time delay due to visual interference) vary across the lifespan as the first power of age. Linear ML models fit the dependence of these test results on age.

Scatter plot Figure 7A reports the linear distribution of the results in the RMO test over the years of life. After 20, reaction time in the RMO test is not affected by age. A suggested explanation for this comes from a completely different paradigm of testing RMO compared to the other tasks. During the RMO test, the examinee is asked to respond to events that happen at a prearranged moment in time. Before taking the other tests, the individual is instructed to wait for an unexpected event to occur (e.g., an appearance of a targeted stimulus in ASR and IRT or a light flash in SVMR and CVMR tests). But the accuracy in the RMO test changes with age. For this reason, the variance of reaction time (RMO_variance) follows a green parabolic trendline in Figure 7B with the best performance in the age range from 35 to 40 years.

**Figure 8:**
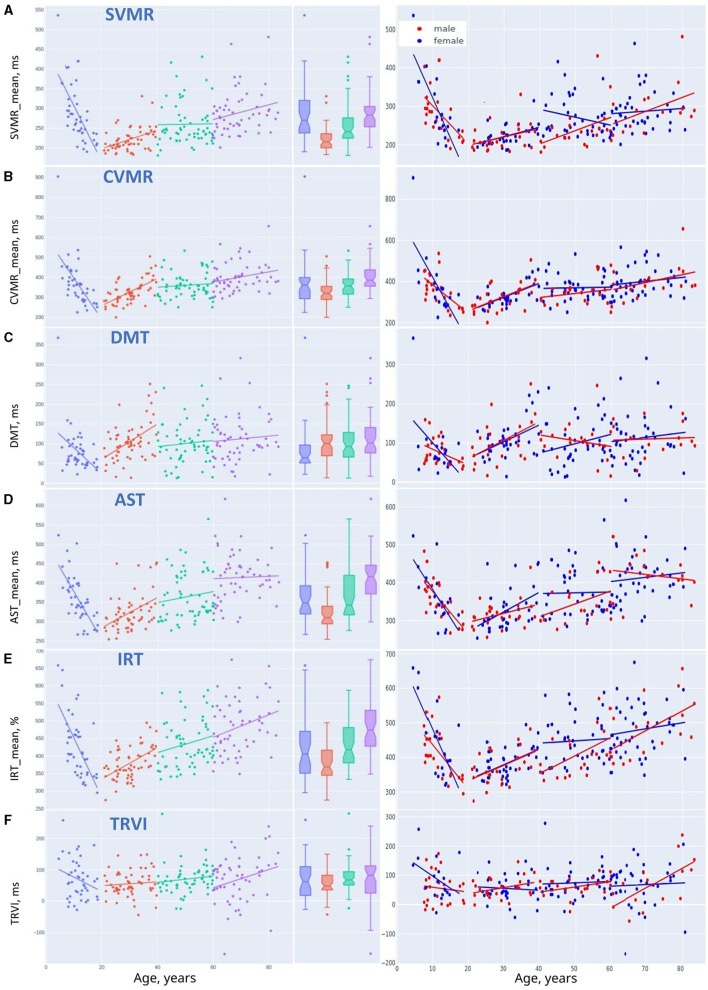
Linear trendlines of performance in psychophysiological tests in age groups: **(A)** SVMR_mean, **(B)** CVMR_mean, **(C)** DMT, **(D)** AST_mean, **(E)** IRT_mean, and **(F)** TRVI.

Linear regression trendlines in Figures 7C, 8 indicate changes in psychophysiological performance for each age group. From the diagrams, it is seen that the results in psychophysiological tests follow a common age-related tendency. After 20, the performance starts worsening with advancing age.

### 4.2. Sex Differences in Lifelong Dynamics of Brain Morphometry

The data in Table 3 justify the equal distribution of female and male study participants over age. This enables us to compare their brain morphometric data.

**Table 3:** Demographics of subjects.

**Age groups**,	**Total**	**Male**	**Female**
**years**	**(m/f)**	**mean ±Std**	**mean ±Std**
4–9	14(10/4)	8.53 ± 0.66	6.22 ± 1.52
10–20	29(15/14)	14.08 ± 2.9	12.78 ± 1.88
20–30	30(14/16)	25.03 ± 2.81	26.88 ± 2.38
30–40	28(9/19)	35.33 ± 2.25	34.3 ± 3.54
40–50	30(9/21)	44.69 ± 3.49	46.12 ± 2.99
50–60	29(12/17)	56.42 ± 2.96	56.21 ± 2.63
60–70	36(7/29)	65.04 ± 3.73	64.92 ± 3.2
70+	19(8/11)	78.58 ± 4.23	75.53 ± 3.4

Table 1 contains the data for both sexes. In the table, every age group has *p*-values that reflect the differences between females and males. For absolute volumes of brain compartments (e.g., GM, WM, CSF, etc.). Mann-Whitney *U*-test provided a significant difference (*p* < 0.05) between sexes. There is much evidence that the skull size accounts for the major part of these findings as the difference between sexes in TIV was the most prominent (*p* ≤ 0.002) if compared to the brain parts.

Adjusted to the skull volume, the relative volumes of the brain compartments (e.g., GM%, WM%, CSF%, etc.) do not differ markedly for both sexes in Adolescents and Young adults. The tendency changes after 40 years of age. The percentage of total GM is prominently higher in women in Middle-aged individuals (31.85 ± 3.96 vs. 29.46 ± 2.86; *p* = 0.009) and in Older adults (30.94 ± 3.65 vs. 28.87 ± 2.38; *p* = 0.0145). The data for cortical GM differ substantially between sexes in the Midlife adults (21.64 ± 3.68 vs. 19.54 ± 2.91; *p* = 0.0142). This can be explained by the sex differences in the speed and/or in the onset of atrophic changes in GM. Atrophy either starts earlier or goes faster in males. Atrophy of GM also accounts for a pronounced difference in GM-to-WM ratio between sexes in the Midlife adults (77.43 ± 15.81 vs. 70.17 ± 14.65; *p* = 0.0375) The tendency is near to significant in Older adults (76.18 ± 17.77 vs. 68.22 ± 12.14; *p* = 0.0741)

A marked difference in the proportion of total CSF (22.68 ± 3.33 vs. 26.79 ± 4.94; *p* = 0.0036) and iCSF (1.75 ± 0.79 vs. 2.54 ± 1.02; *p* = 0.0033) shows that elderly men are much more prone to age-related atrophy than women of the same age. The difference is also profound in the iCSF-to-CSF ratio after the age of 60 years (7.59 ± 2.72 vs. 9.25 ± 2.7; *p* = 0.0076) The distribution of the WMH-to-WM index has its sex-related features that are near to significant in the age range from 40 to 60 years (2.76 ± 1.37 vs. 3.22 ± 1.19; *p* = 0.0595) and after the age of 60 (2.58 ± 1.51 vs. 3.04 ± 1.1; *p* = 0.0511).

### 4.3. Mathematical Models of Age-Related Changes

As mentioned before, the ridge regression model applied to the linear and non-linear function of age (refer to Equations 9, 10) allowed us to build two types of approximation functions for various attributes. Those types are a straight line and a parabola (second-order line). We supplied scatter plots with trendlines for linear and polynomial kernel non-linear models and their 95% CI in Figures 2, 4–7. This improved visual selection of the best fitting model.

From the diagrams for voxel-based morphometry data in Figures 2, 4A–D, the data are scattered across the lifespan more than the psychophysiological variables. For this reason, it is hard to select a proper mathematical model using a visual trajectory of the life-long changes.

The scatter plots for the psychophysiological tests (Figures 5–7) show that the data can be grouped into two categories. *The first category* contains the variables of reaction time in SVMR, CVMR, AST, and IRT tests and variability of the time in all the tests, including RMO. This category has a U-shaped form of distribution over age and the polynomial kernel regression model fits it better. *The second category* comprises the variables that reflect task switching (DMT, TRVI) and the balance of processes in the central nervous system (RMO_mean). The linear models reflect changes of these variables reliably.

#### 4.3.1. Performance of the Linear and Non-linear Models

To justify the selection of preferable mathematical models, we compared the potential of the models to predict the structural and functional changes in the brain. For this, we calculated performance metrics (refer to Table 4). As the association of the aforementioned variables with age is statistically significant (*p* < 0.05), the number of years can be used as a predictor in the regression models.

**Table 4:** Performance of linear and non-linear ML regression models using age to predict results of psychophysiological tests.

	**Linear regression model**				**Polynomial kernel regression model**				**Performance gain**
	**MAE**	**RMSE**	**R2**	** $\frac{MAE}{range},\%$ **	**MAE**	**RMSE**	**R2**	** $\frac{MAE}{range},\%$ **	
**Voxel-based morphometry**									
GM, %	2.78	13.82	0.41	**11.19**	2.72	12.89	0.45	**10.95**	**0.24**
cortical GM / GM	2.81	13.58	0.18	**12.2**	2.68	12.5	0.24	**11.64**	**0.56**
WM, %	4.2	31.24	0.14	**11.74**	4.19	30.22	0.17	**11.74**	**0.00**
WMH / WM	0.92	1.46	0.003	**13.3**	0.91	1.45	0.005	**13.2**	**0.10**
CSF, %	2.06	7.56	0.64	**7.8**	1.99	7.2	0.66	**7.54**	**0.26**
intraventricular	1.78	6.58	0.1	**10.6**	1.75	6.44	0.12	**10.39**	**0.21**
CSF / total CSF									
**Psychophysiological tests**									
SVMR_mean	43.65	3466.29	0.02	**12.11**	39.53	2890.73	0.18	**10.97**	**1.14**
CVMR_mean	55.39	6068.92	0.06	**7.87**	53.03	5585.68	0.13	**7.54**	**0.33**
DMT	38.32	2786.15	0.04	**10.80**	38.14	2782.11	0.04	**10.75**	**0.05**
AST_mean	49.67	3745.09	0.14	**13.66**	45.90	3382.66	0.22	**12.63**	**1.03**
IRT_mean	58.21	5414.28	0.13	**14.53**	54.53	4697.82	0.25	**13.61**	**0.92**
TRVI	40.92	3089.75	0.01	**9.15**	40.15	3030.01	0.03	**8.98**	**0.17**
RMO_mean	45.26	6237.70	0.00	**4.73**	45.22	6236.17	0.00	**4.73**	**0.00**

*MAE/range values and difference between them are marked in bold font*.

The low R-squared (*R*^2^) values of the prediction models indicate the high variability around the regression line. This can be explained by the nature of our data: the psychophysiological performance is unstable, and it reflects the adjustment of an individual to the living conditions (Habuza et al., 2021b,c; Statsenko et al., 2021b). Nonetheless, the reproducibility of the PTs and their informative value allows us to consider the tests as a tool for screening psychological misadjustment and cognitive decline (Statsenko and Charykova, 2010).

The variables with the observed quadratic trendlines (SVMR_mean, CVMR_mean, AST_mean, IRT_mean) show some increase in the accuracy of prediction obtained with the second-degree polynomial function of age. The bigger the curvature of the parabola is, the larger the dissemblance between the performance of the linear and non-linear models will be. The performance metric that we use to compare different models is the proportion of MAE to the range of the studied values (MAE/range). One may rank the models with regard to the difference in the performance of the models (refer to Table 5).

**Table 5:** Psychophysiological and morphological variables ranked with regard to distance between performance of linear and quadratic models.

**Psychophysiological performance**		**Morphological variables**	
**Distance**	**Variable**	**Distance**	**Variable**
1.14	SVMR_mean	0.56	cGM / GM
1.03	AST_mean	0.26	CSF%
0.92	IRT_mean	0.24	GM%
0.33	CVMR_mean	0.21	iCSF / CSF
0.17	TRVI	0.1	WMH / WM
0.05	DMT	0	WM%
0	RMO_mean		

In the left column of Table 5, we put a list of psychophysiological variables ranked according to the difference in the performance of the linear and non-linear models: SVMR_mean, AST, IRT, CVMR, TRVI, DMT, and RMO. The variables at the top of the list reflect a cognitive domain named *informative processing speed*. The life-long changes in the tests match polynomial trendlines much better than the linear ones. The variables at the bottom of the list (*DMT*, *TRVI*) reflect the performance of another cognitive subdomain which is *task switching and inhibitory control*. Their age-related distribution almost fits the linear model. Both the first and second-degree polynomial function models for *RMO*_*mean* have equal performance, because the results of the RMO test do not depend on age.

In the left column of Table 5, we ranked morphological variables with regard to the distance between the performance of the models: cGM/GM, CSF%, GM%, iCSF/CSF, WMH/WM, and WM%. In general, age-related changes of total GM (*GM%*) and its cortical part (*cGM*/*GM*) follow the quadratic trend of retardation. The same is true for the total CSF (*CSF%*) and the portion of its intraventricular part (*iCSF*/*CSF*). One may use linear models to describe the distribution of total WM (*WM%*) and its lesions (*WMH*/*WM*) over age because a quadratic equation does not provide an advanced performance.

### 4.4. Comparison of Brain Structural Changes With Dynamics of Psychophysiological Performance

#### 4.4.1. Association Between Brain Volumetric Data and Functional Outcomes Throughout Life

**Figure 9:**
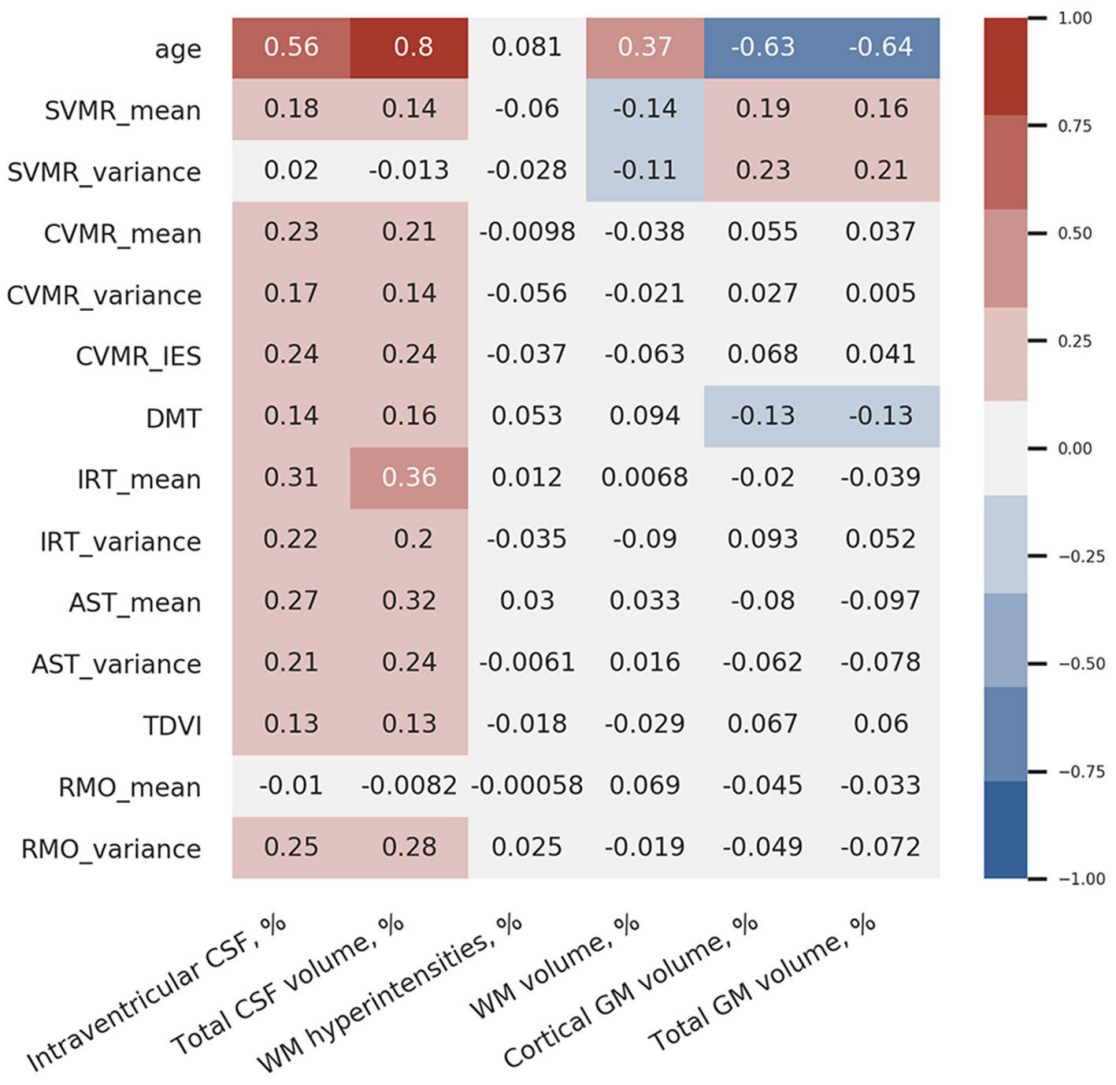
Correlation coefficients between brain volumetric data and psychophysiological performance.

Figure 9 shows the coefficients of correlation between the brain volumetric data and the major dependent variables of the battery of PTs used. From this diagram, there is a strong positive association between the total volume of CSF and the latency of reacting in SVMR, CVMR, IRT, AST tests. A positive dependence is also observed in the volume of brain ventricles and reaction time in the tests. Both CSF% and iCSF% positively correlate with age (*R*-values are 0.8 and 0.56 correspondently). This justifies the indices as markers of age-related brain atrophy. As the correlation between CSF% and age is the strongest one (maximal *r*-value), the latter can be considered to be the most sensitive marker of the atrophy related with age.

Interestingly, the strongest correlation between the brain structural data and the functional outcomes is observed between total CSF% and reaction time in the IRT that is most cognitively demanding in our battery (*r* = 0.36). The test employs a set of cognitive domains and subdomains such as information processing, switching and inhibitory control, attention.

We also found that the variance of reaction time in the SVMR test (SVMR_variance) is not associated with age; it demonstrates a negative association with the relative volume of WM (WM%; *r* = −0.11). There is a moderate positive association between SVMR_variance and the relative volume of GM% (*r* = 0.21), particularly the GM cortical part (cGM%; *r* = 0.23).

Simple visual-motor reaction test is the easiest test in our battery as it utilizes only information processing with a minimal load on other cognitive functions. The mean reaction time in this test (SVMR_mean) has a moderate positive association with the relative volumes of the CSF (for CSF% *r* = 0.14; for iCSF% *r* = 0.18) and GM (for GM% *r* = 0.16; for cGM% *r* = 0.19). In addition, SVMR_mean has a moderate negative statistical relationship with the proportion of WM% (*r* = −0.14). A better brain connectivity in individuals with a high proportional volume of total WM may account for the faster reaction. The association is direct in the SVMR test because it does not require decision-making as in “go/no-go” test, and it does not employ attention as AST and IRT tests do. For this reason, the association with the brain connectivity is straight-forward in this test.

Decision-making time is positively associated with the relative volumes of the CSF (for CSF% *r* = 0.16; for iCSF% *r* = 0.14) and negatively with the relative volumes of GM (*r* = 0.13 for both GM% and cGM%). These facts prove the reliability of the PTs used because decisions are made in the GM cortex. Despite the validity of the tests, the weak associations (*r* ≤ 0.16) limit the utility of DMT as a biomarker of brain atrophy changes.

There is no association between the percentage of WM lesions (WMH%) and psychophysiological performance; as well as no correlation is seen between WMH% and age. The mean reaction time in the RMO test (RMO_mean) is associated neither with age nor with the volume of the major brain compartments.

#### 4.4.2. Comparison of Slopes for Brain Structural and Functional Changes in Age Groups

Table 6 provides a comparison of the slopes for psychophysiological performance with those for the structural brain changes. In all age periods, except for midlife, the dynamics of the tests results markedly differ from those for brain volumetric data. The number of significant differences between the slopes is the highest in Adolescents, moderate in Young adults and the lowest in Older adults. The supposed explanations for this are the following. Massive neurodevelopment accounts for the disproportional changes in the brain structure and cognitive functioning in Adolescents. A continuous accumulation of skills and educational experience justifies the same tendency in Young adults. Due to the ongoing process of acquiring knowledge till roughly the age of over 40 years, the cognitive performance of individuals may still improve at the time when irreversible structural decline is already taking place. Dramatic changes in the brain compartments and cognitive performance lead to substantial differences in the dynamics of structural atrophy and functional decline.

**Table 6:** Difference of slopes for brain structural data and mean reaction time in psychophysiological tests.

	***Adolescent***			***Young adults***			***Midlife adults***			***Older adults***		
	**Estimate ± Std**	**CI**	**p**	**Estimate ± Std**	**CI**	**p**	**Estimate ± Std**	**CI**	**p**	**Estimate ± Std**	**CI**	**p**
**SLOPE DIFFERENCE BETWEEN PSYCHOPHYSIOLOGICAL PARAMETERS AND MORPHOLOGICAL BRAIN ESTIMATES**												
*SVMR vs. TIV*	35.9 ± 6.58	[22.6;48.8]	**5.71e-07**	–7.386 ± 5.444	[–18.173;3.401]	0.178	0.512 ± 3.72	[–6.86;7.88]	0.891	5.3 ± 3.51	[–1.66;12.3]	0.134
*SVMR vs. CSF*	19.249 ± 2.899	[13.5;25.0]	**3.17e-09**	–0.295 ± 1.49	[–3.25; 2.66]	0.844	1.39 ± 1.78	[–2.13;4.9]	0.435	–6.74 ± 1.82	[–10.3;–3.13 ]	**0.000344**
*SVMR vs. CSFi*	14.3708 ± 2.53	[9.33;19.3]	**2.06e-07**	–7.39 ± 5.44	[–18.2;3.4 ]	0.178	0.148 ± 1.22	[–2.26;2.56 ]	0.903	–0.744 ± 1.13	[–2.99;1.5 ]	0.513
*SVMR vs. GM*	13.5 ± 3.31	[6.94;20.1]	**0.000102**	–6.69 ± 2.01	[–10.7; –2.7]	**0.001206**	–0.63 ± 1.79	[-4.17;2.91]	0.725	–3.49 ± 1.47	[–6.4;–0.571 ]	**0.0196**
*SVMR vs. cGM*	11.9 ± 3.03	[5.83;17.9]	**0.000187**	–5.55 ± 1.8	[–9.12; –1.99]	**0.00257**	–0.588 ± 1.71	[–3.98;2.81 ]	0.732	–3.22 ± 1.4	[–6;–0.443 ]	**0.023**
*SVMR vs. WM*	29.1 ± 3.94	[21.3;37.0]	**1.1e-10**	–3.10 ± 3.22	[–9.47;3.17 ]	0.337	0.177 ± 2.5	[–4.78;5.13]	0.944	4.05 ± 2.53	[–0.972;9.07 ]	0.113
*SVMR vs. WMH*	14.1 ± 2.52	[9.12;19.1]	**2.66e-07**	–2.49 ± 0.74	[–3.95;–1.02 ]	**0.00106**	–0.091 ± 1.22	[–2.5;2.32]	0.94	–1.65 ± 1.12	[–3.86;0.567]	0.143
*SVMR vs. GM/WM*	11.25 ± 2.56	[6.15;16.4]	**3.41e-05**	–2.85 ± 0.841	[–4.52;–1.18 ]	**0.000979**	–0.216 ± 1.24	[–2.66;2.23]	0.862	–2.89 ± 1.13	[–5.14;–0.645 ]	**0.012**
*DMT vs. TIV*	28.2 ± 6.49	[15.2;41.1]	**2.2e-16**	–9.53 ± 5.51	[–20.5;1.4 ]	0.087	–0.147 ± 3.71	[–7.49;7.2]	0.968	6.48 ± 3.55	[–0.558;13.5 ]	0.071
*DMT vs. CSF*	11.689 ± 2.689	[6.34; 17.0]	**3.93e-05**	–2.44 ± 1.73	[–5.86;0.989 ]	0.161	0.731 ± 1.75	[–2.74; 4.20 ]	0.677	7.91 ± 1.89	[4.17;11.7 ]	**5.82e-05**
*DMT vs. CSFi*	6.81 ± 2.29	[2.25; 11.4]	**0.00387**	–4.24 ± 1.13	[–6.48;–2.01 ]	**0.000265**	–0.511 ± 1.18	[–2.85;1.82]	0.666	0.432 ± 1.24	[–2.02;2.89]	0.728
*DMT vs. GM*	5.966 ± 3.13	[–0.257;12.2]	0.06	–8.84 ± 2.2	[–13.2;–4.48 ]	**0.000105**	–1.29 ± 1.76	[–4.78; 2.2]	0.466	–2.31 ± 1.55	[–5.39;0.77 ]	0.14
*DMT vs. cGM*	4.3 ± 2.83	[–1.33;9.93]	0.133	–7.7 ± 2.0	[–11.7; –3.73 ]	**0.000201**	1.25 ± 1.69	[–2.1; 4.59]	0.462	–2.04 ± 1.49	[–5; 0.906]	0.172
*DMT vs. WM*	21.561 ± 3.788	[14.0; 29.1]	**1.89e-07**	–5.24 ± 3.33	[–11.8; 1.36 ]	0.119	–0.482 ± 2.48	[–5.4; 4.44]	0.847	5.23 ± 2.58	[0.106; 10.3]	**0.046**
*DMT vs. WMH*	6.57 ± 2.27	[2.05; 11.1]	**0.004926**	–4.63 ± 1.15	[–6.9; –2.36 ]	**9.94e-05**	–0.75 ± 1.18	[–3.08; 1.58]	0.526	–0.47 ± 1.22	[–2.9;1.96 ]	0.702
*DMT vs. GM/WM*	3.69 ± 2.33	[–0.936;8.32]	0.116392	–4.99 ± 1.22	[–7.4; –2.58 ]	**4.751e-07**	–0.874 ± 1.2	[–3.25;1.5 ]	0.467	–1.71 ± 1.24	[–4.17; 0.742]	0.169
*AST vs. CSF*	17.632 ± 2.493	[12.672;22.592]	**4.66e-10**	–1.863 ± 1.724	[–5.279;1.554]	0.28232	0.084 ± 1.879	[–3.639; 3.806]	0.965	8.293 ± 1.932	[4.462;12.124]	**3.93e-05**
*AST vs. iCSF*	12.754 ± 2.057	[8.661; 16.847]	**2.18e-08**	–3.668 ± 1.118	[–5.884;–1.452]	**0.00138**	–1.158 ± 1.364	[–3.859;1.544]	0.398	0.814 ± 1.305	[–1.774;3.401]	0.534
*AST vs. GM/WM*	9.632 ± 2.096	[5.463; 13.802]	**1.55e-05**	–4.417 ± 1.208	[–6.810;–2.024]	**0.00039**	–1.521 ± 1.381	[–4.256;1.214]	0.27283	–1.333 ± 1.304	[–3.919;1.253]	0.30925
*AST vs. GM*	11.909 ± 2.962	[6.017; 17.801]	**0.000128**	–8.261 ± 2.193	[–12.606;–3.916]	**0.000265**	–1.936 ± 1.889	[–5.679;1.807]	0.3077	–1.93 ± 1.607	[–5.116;1.257]	0.2325
*AST vs. cGM*	10.242 ± 2.645	[4.98;15.503]	**0.000216**	–7.12 ± 1.997	[–11.078;–3.162]	**0.000537**	–1.893 ± 1.821	[-5.502;1.715]	0.301	-1.663 ± 1.543	[–4.722;1.397]	0.284
*AST vs. TIV*	34.106 ± 6.415	[21.344; 46.868]	**8.96e-07**	–8.954 ± 5.513	[–19.877;1.968]	0.1071	–0.794 ± 3.77	[–8.263;6.675]	0.834	6.859 ± 3.572	[–0.223;13.942]	0.05753
*AST vs. WM*	27.503 ± 3.651	[20.239; 34.767]	**5.86e-11**	–4.669 ± 3.331	[–11.269;1.931]	0.16377	–1.128 ± 2.576	[–6.231;3.974]	0.66213	5.609 ± 2.615	[0.424;10.793]	**0.03425**
*AST vs. WMH*	12.516 ± 2.039	[8.459;16.573]	**2.85e-08**	–4.056 ± 1.139	[–6.314;–1.798]	**0.000546**	–1.397 ± 1.363	[–4.097;1.303]	0.307594	–0.088 ± 1.29	[–2.646;2.470]	0.946
*CVMR vs. CSF*	25.791 ± 4.213	[17.411;34.171]	**3.03e-08**	–4.783 ± 1.769	[–8.287;–1.278]	**0.00792**	0.604 ± 1.893	[–3.146;4.353]	0.750	6.085 ± 2.057	[2.007;10.162]	**0.00381**
*CVMR vs. iCSF*	20.913 ± 3.97	[13.015; 28.811]	**1.09e-06**	–6.588 ± 1.186	[–8.937;–4.239]	**1.88e-07**	–0.638 ± 1.383	[–3.377;2.101]	0.646	–1.394 ± 1.483	[–4.335;1.546]	0.34918
*CVMR vs. GM/WM*	17.791 ± 3.990	[9.853; 25.729]	**2.59e-05**	–7.337 ± 1.27	[–9.854;–4.82]	**6.97e-08**	–1.001 ± 1.399	[–3.773;1.771]	0.47572	–3.541 ± 1.482	[–6.480;–0.602]	**0.01868**
*CVMR vs. GM*	20.068 ± 4.506	[11.104; 29.031]	**2.64e-05**	–11.181 ± 2.228	[–15.595;–6.766]	**1.98e-06**	–1.416 ± 1.903	[–5.186;2.354]	0.4584	–4.138 ± 1.755	[–7.617;–0.659]	**0.0202**
*CVMR vs. cGM*	–18.401 ± 4.304	[–26.963; –9.839]	**5.12e-05**	10.04 ± 2.036	[6.006;14.074]	**2.85e-06**	1.373 ± 1.836	[–2.263;5.010]	0.456	3.871 ± 1.696	[0.508;7.234]	**0.0245**
*CVMR vs. TIV*	42.265 ± 7.258	[27.825; 56.704]	**1.09e-07**	–11.874 ± 5.527	[–22.825;–0.924]	**0.0338**	–0.274 ± 3.777	[–7.756;7.209]	0.9423	4.651 ± 3.641	[–2.568;11.870]	0.20424
*CVMR vs. WM*	35.662 ± 4.986	[25.74;45.582]	**3.25e-10**	–7.589 ± 3.354	[–14.235;–0.943]	**0.02559**	–0.608 ± 2.586	[–5.731;4.514]	0.81438	3.4 ± 2.708	[–1.969;8.769]	0.212
*CVMR vs. WMH*	20.675 ± 3.961	[12.795; 28.555]	**1.33e-06**	–6.976 ± 1.205	[–9.364;–4.588]	**6.61e-08**	–0.877 ± 1.382	[–3.614;1.861]	0.527	–2.296 ± 1.47	[–5.211;0.618]	0.12124
*IRT vs. CSF*	22.186 ± 3.387	[15.447; 28.924]	**4.73e-09**	–2.472 ± 1.671	[–5.783;0.838]	0.141768	–0.896 ± 1.968	[–4.794;3.001]	0.6495	5.375 ± 2.09	[1.231;9.518]	**0.01151**
*IRT vs. iCSF*	–17.307 ± 3.081	[–23.436;–11.179]	**2.58e-07**	4.278 ± 1.034	[2.229;6.327]	**6.83e-05**	2.138 ± 1.483	[–0.801;5.076]	0.152248	2.104 ± 1.529	[–0.927;5.135]	0.17156
*IRT vs. GM/WM*	–14.186 ± 3.107	[–20.366; –8.006]	**1.73e-05**	5.027 ± 1.13	[2.787;7.266]	**2.06e-05**	2.501 ± 1.499	[–0.468;5.470]	*0.09787*	4.251 ± 1.528	[1.221;7.281]	**0.00801**
*IRT vs. GM*	16.462 ± 3.746	[9.011; 23.914]	**3.29e-05**	–8.87 ± 2.151	[–13.132;–4.608]	**7.19e-05**	–2.916 ± 1.977	[–6.833;1.001]	0.1431	–4.848 ± 1.794	[–8.404;–1.292]	**0.00801**
*IRT vs. cGM*	–14.795 ± –14.795	[–21.759; –7.832]	**6.12e-05**	7.73 ± 1.951	[3.863;11.596]	**0.000132**	2.873 ± 1.913	[–0.916;6.662]	0.136	4.581 ± 1.737	[1.138;8.024]	**0.0096**
*IRT vs. TIV*	38.659 ± 6.813	[25.106;52.212]	**2.03e-07**	–9.564 ± 5.496	[–20.454;1.326]	*0.0846*	–1.774 ± 3.815	[–9.332;5.784]	0.6428	3.941 ± 3.66	[–3.315;11.198]	0.28398
*IRT vs. WM*	32.057 ± 4.312	[23.480;40.634]	**9.10e-11**	–5.279 ± 3.304	[–11.824;1.267]	0.112902	–2.109 ± 2.641	[–7.340;3.123]	0.42630	2.69 ± 2.733	[–2.729;8.110]	0.3272
*IRT vs. WMH*	17.07 ± 3.069	[10.965; 23.174]	**3.25e-07**	–4.666 ± 1.057	[-6.759;–2.572]	**2.34e-05**	–2.377 ± 1.482	[–5.313;0.560]	0.111626	–3.006 ± 1.516	[–6.012;0]	**0.04999**
*RMO vs. CSF*	11.490 ± 3.427	[4.673;18.307]	**0.00121**	2.447 ± 1.951	[–1.420;6.313]	0.212518	2.836 ± 2.674	[–2.461;8.132]	0.291	6.166 ± 2.118	[1.967;10.365]	**0.00439**
*RMO vs. iCSF*	–6.612 ± 3.124	[–12.826; –0.397]	**0.03730**	–0.641 ± 1.444	[–3.502;2.22]	0.658	–1.594 ± 2.341	[–6.231;3.042]	0.497	1.313 ± 1.567	[–1.793;4.420]	0.404
*RMO vs. GM/WM*	–3.491 ± 3.149	[–9.755; 2.774]	0.27090	0.108 ± 1.514	[–2.893;3.108]	0.94342	–1.231 ± 2.350	[–5.887;3.426]	0.602	3.46 ± 1.566	[0.354;6.565]	**0.02935**
*RMO vs. GM*	5.767 ± 3.781	[–1.755;13.289]	0.13110	–3.951 ± 2.375	[–8.658;0.755]	*0.099*	0.816 ± 2.681	[–4.495;6.127]	0.76138	–4.057 ± 1.826	[–7.677;-0.436]	**0.0285**
*RMO vs. cGM*	–4.100 ± 3.538	[–11.139;2.939]	0.25000	2.811 ± 2.196	[–1.541;7.162]	0.2033	–0.859 ± 2.634	[–6.076;4.359]	0.744984	3.79 ± 1.77	[0.280;7.299]	**0.2091**
*RMO vs. TIV*	27.964 ± 6.832	[14.372;41.555]	**9.92e-05**	–4.645 ± 5.588	[–15.717;6.426]	0.408	1.958 ± 4.223	[–6.408;10.324]	0.644	4.733 ± 3.676	[–2.556;12.021]	0.2091
*RMO vs. WM*	21.362 ± 4.343	[12.723;30.000]	**4.41e-06**	–0.36 ± 3.454	[–7.203;6.484]	0.917	1.623 ± 3.202	[–4.720;7.967]	0.613130	3.482 ± 2.755	[–1.980;8.944]	0.2091
*RMO vs. WMH*	6.374 ± 3.112	[0.183; 12.565]	**0.04373**	0.253 ± 1.46	[-2.64;3.147]	0.863	1.355 ± 2.34	[-3.280;5.991]	0.564	-2.215 ± 1.555	[-5.297;0.867]	0.1572
*TRVI vs. CSF*	9.701 ± 3.155	[3.424; 15.978]	**0.00286**	1.44 ± 1.672	[–1.873;4.754]	0.390867	0.536 ± 1.648	[–2.729;3.801]	0.7456	5.695 ± 2.085	[1.561;9.829]	**0.00739**
*TRVI vs. iCSF*	-4.823 ± 2.823	[-10.440;0.794]	0.09141	0.365 ± 1.036	[–1.688;2.418]	0.725	0.705 ± 1.022	[–1.320;2.731]	0.492	1.784 ± 1.522	[–1.233;4.802]	0.244
*TRVI vs. GM/WM*	-1.701 ± 2.852	[–7.374;3.971]	0.552	1.114 ± 1.132	[-1.129;3.357]	0.3273	1.069 ± 1.045	[-1.001;3.139]	0.3086	3.93 ± 1.521	[0.914;6.947]	**0.0111**
*TRVI vs. GM*	3.978 ± 3.537	[–3.059;11.014]	0.264054	-4.958 ± 2.152	[–9.222;-0.693]	**0.0231**	–1.483 ± 1.66	[–4.772;1.805]	0.373	–4.528 ± 1.788	[–8.072;-0.983]	**0.0128**
*TRVI vs. cGM*	–2.311 ± 3.276	[–8.828; 4.207]	0.483	3.817 ± 1.953	[–0.052;7.686]	*0.0531*	1.441 ± 1.582	[–1.694;4.575]	0.364	4.261 ± 1.731	[0.830;7.691]	**0.0154**
*TRVI vs. TIV*	26.175 ± 6.7	[12.845; 39.504]	**0.000192**	–5.651 ± 5.497	[–16.542;5.240]	0.306	–0.341 ± 3.661	[–7.593;6.911]	0.926	4.262 ± 3.657	[–2.989;11.513]	0.247
*TRVI vs. WM*	19.572 ± 4.132	[11.353; 27.792]	**8.98e-06**	–1.366 ± 3.304	[–7.913;5.181]	0.680	–0.676 ± 2.413	[–5.455;4.103]	0.780	3.011 ± 2.73	[–2.401;8.423]	0.2725
*TRVI vs. WMH*	4.585 ± 2.810	[–1.006;10.176]	0.10662	–0.753 ± 1.059	[–2.851;1.345]	0.479	–0.944 ± 1.021	[–2.967;1.079]	0.357	–2.686 ± 1.509	[–5.678;0.307]	0.07802

*Structural features are reported in % of TIV. Significant differences between slopes (p < 0.05) are marked in bold font. CI - 95% confidence interval*.

## 5. Discussion

### 5.1. Lifelong Changes of Major Brain Compartments

The number of studies devoted to neuroimages of degenerative disease is disproportionally higher than the same type of research onto normal brain aging. To describe clinical populations, neurobiologists and neurophysiologists focus on the changes in either the structure or the activity of the brain. A dearth of information concerning baseline morphology and physiology of the “normal” brain during aging makes interpretation of clinical data even more challenging (Weyandt et al., 2013). To improve the situation, we tried to make inferences regarding natural dynamics of the brain structure and function in a healthy population.

#### 5.1.1. Skull Morphometry

Age-related changes of TIV were an issue of studies in the past, and they still remain under discussion. The results of the studies may differ because of inconsistency in the following settings: the study design (e.g., cross-sectional or longitudinal), selected population, methodology, etc. This was evidenced in a cross-sectional study of people born within a time interval of 40 years. In that study an average TIV was larger in the younger examinees. In the observation the skull size closely correlated with the height of the individuals (Kim et al., 2018). In another cross-sectional study of people aged from 24 to 80 years, TIV did not vary among different generation. Supposedly, the results of the studies are inconsistent due to the social-economic factors that may account for the discrepancies (González-José et al., 2005; Bouthoorn et al., 2012).

Our study had a cross-sectional design. It found the largest mean value of TIV in Adolescents. This justifies that an increase in skull volume is consistent with the general trend toward enlargement of the human body in subsequent generations.

#### 5.1.2. Subarachnoid Space

In recent studies, an increase in the ratio of the CSF compartment to the brain parenchyma was linked with cerebral atrophy in aging (Coffey et al., 1992). An outcome of the increase in CSF compartment is a reduction of liquor turnover from four-five times to three times a day (Sakka et al., 2011). Replacement time for the enlarged volume is longer (Preston, 2001).

In the elderly, the turnover of CSF reduces for several reasons. The first reason is, as mentioned before, *an increased volume of subarachnoid space*. The second reason is a *reduced production of CSF by the choroid plexus*. The production decreases by nearly two times in humans and animals (Chiu et al., 2012). The final reason is *a reduction in the capacity of lymphatic outflow pathways to filter both large and small molecules in older age* (Ma et al., 2017). In a study of changes in the brain morphology from infancy to late adulthood, sulcal CSF volume remained stable to about the age of 20 years, then showed a curvilinear increase throughout adulthood. The acceleration was higher after 50 years (Pfefferbaum et al., 1994). It is plausible that the increase in CSF volume in aging reflects atrophic processes such as cell shrinkage. According to another study, the ratio of sulcal CSF volume to intracranial volume was higher in the elderly aged 55 years and above than younger examinees (Gur et al., 1991). Others reported a linear reduction in the brain volume and an increase in CSF (Jernigan et al., 1990).

A previous study looked into the global and regional effects of age on CSF volume of individuals between 18 and 79 years. Researchers observed a gradual increase in the volume of CSF at a rate of *R*^2^ = 0.377. The same was true for the CSF parts allocated between sulci and inside ventricles. In regional effects of age, researchers found a relatively little enlargement of the CSF space in the pontine cistern, including its caudal extent around the medulla. The highest symmetrical increase in the CSF space was seen in the following locations: chiasmatic and supracerebellar cisterns, cisterna magna, third ventricle, the Sylvian and interhemispheric fissures. The regional effects tended to be *linear*. The fit of the data did not improve when they used the second and third-order polynomial expansion of age. They did not find evident interaction of the pCSF volume with sex either globally or in the regional effects (Good et al., 2001). Among the elderly volunteers aged 60–84, a nonplanimetric technique of measuring intracranial CSF volume showed a strong correlation between the CSF and age but a weak correlation between CSF and TIV. This observation proved that the normal brain volume decreases with time (Malko et al., 1991).

#### 5.1.3. Brain Ventricles

Studying the brain of healthy individuals has enabled researchers to get a new insight into a neurobiological foundation for age-related cognitive changes and their impact on cognitive function. The majority of previous reports indicate that brain volume reduces and the volume of the ventricles enlarges with time, which suggests that brain atrophy in humans is associated with senescence. Age-related ventricular enlargement is presumed to occur as a result of shrinkage of periventricular brain matter (Kwon et al., 2014). Other researchers confirmed this conclusion with a CT assessment (Earnest et al., 1979). To do this, they monitored the ventricles of a group of normal individuals aged 60–99 years. They observed a more prominent enlargement between 80 and 99 years. This supports the findings of other researchers that the enlargement of the lateral ventricles is the highest in the ninth decade. From their observation, the volume increased evenly from the first to the seventh decade and rose relatively higher during the eighth and ninth decades (Barron et al., 1976). It explains why a malformation of the ventricular volume is easier to identify in younger individuals.

According to the data from other studies, subjects of two age groups (16–40 and above 60 years) had different sizes of ventricles and of sulci. In the elderly, the ventricles were enlarged (Preul et al., 2006). An MRI-based study of the lifelong dynamics of lateral ventricles and CSF volume confirmed the previous CT findings. The ventricular volume increased linearly until the age of 40 with a dramatic rise after 60 (Foundas et al., 1998).

Authors agree that the rate of change in ventricular size may be of considerable interest in longitudinal analyses. In a longitudinal study of people from 31 to 84 years, the mean rate of enlargement of ventricles equaled 650 *mm*^3^/*y* (Scahill et al., 2003). The expansion of ventricles consistently accelerated after 60 years of age. Some studies observed a marked increase in ventricular, frontal lobe measures with time.

In healthy adult men, between 19 and 92, the right temporal lobe was larger than the left one. This increase in volume of the temporal horn of the lateral ventricle supports the notion of a smaller volume of the hippocampus in the older group (Pfefferbaum et al., 1994). A prior work demonstrated similar changes: ventricular CSF volume seriously increased, with the left and right lateral ventricles showing significant linear and quadratic trends, while the third ventricle and left temporal horn exhibited pronounced linear trends with a 17% variance in volume. Researchers observed a considerable curve increase for the lateral ventricles to be steeper in the older population (Sullivan et al., 1995).

Several investigators defined normal age-specific values for brain morphometrics in healthy men from 21 to 80 years. They found a positive correlation of the volume of CSF with age and reported an elevation of the volumes of the lateral and third ventricles in the elderly (Schwartz et al., 1985). These findings agree with observations on postmortem material where linear measurements in CT scans and pneumoencephalograms demonstrated an increase in the ventricular volumes and an overall growth in CSF volume in the elderly (Barron et al., 1976).

#### 5.1.4. Gray Matter

Research of the past came up with the results that support our findings on a steady decrease in the volume of GM in life.

**Total gray matter**. The GM volume starts decreasing at a very young age and continues changing across adulthood. There is evidence of an increase in the GM volume within 2.5 years from birth. The GM volume expands by 13% from early childhood to the age of 6–9 years. Since then, there is a constant and stable decrease in the GM volume by approximately 5% per decade (Courchesne et al., 2000). Another study of healthy well-developed children (*IQ*−*score* > 80) aged 5–18 years confirmed this. Both absolute and normalized measures of GM increased until 9 years old. This was followed by a decline till the age of 15 and a slight increase thereafter (Wilke et al., 2007). On the contrary, a study of children of 4.5–18 years showed a continuous decline in the GM volume by 6.56 *cm*^3^ per year (Group, 2012).

After 55, the normalized GM volume reduces at an annual rate of 0.183% vs. 2.37*cm*^3^ for the absolute GM volume (Smith et al., 2007). Another study of people aged 59–89 years showed an average 0.40 *cm*^3^ annual loss of the GM volume (Resnick et al., 2000). Evidently, there is no strong agreement on the pace of the changes. Discrepancies in the methodology of the studies may account for such varying findings. A study that combined a longitudinal and cross-sectional design ended up with controversial results. A longitudinal part of the study proved that an insignificant rate of change in the GM volume (*p* > 0.05) remained stable within 3.5 years in people over 35. In contrast to this, a cross-sectional analysis showed a significant association between advancing age and reduction in all brain volumes including GM (Liu et al., 2003).

**Cortical gray matter**. Similar to total GM, absolute cortical GM accumulates and reaches its peak during early childhood. Then the GM volume decreases in the second decade and stabilizes in the third decade (Mills et al., 2016). A study of cortical regions showed that GM atrophy starts in the dorsal parietal cortex and continues in the frontal and temporal cortex (Gogtay et al., 2004).

The current study provides evidence on volumetric changes of GM during aging. Much research has been done on cortical GM atrophy as a factor underlying neurodegenerative disorders and mental diseases. For instance, there is evidence of an accelerated loss of cGM in chronic schizophrenia (Mathalon et al., 2001). Examining cGM, researchers have concentrated mainly on its thickness rather than the volume (Sowell et al., 2004; O'donnell et al., 2005; Koelkebeck et al., 2014; Profant et al., 2020).

#### 5.1.5. White Matter

The pattern of age-related change of the WM volume is different from the one of GM. This is evident from our data and recent studies. However, there is no strong consensus on whether the lesions of WM are characteristic of normal aging or age-related pathology.

**Total white matter**. The WM and GM volumes rise in childhood and at the time of puberty, but the rate of change is different. The GM volume increases by 13% while the WM volume changes by 74% before 12–15 years. Starting from adolescence, the WM volume increases less markedly than in childhood, and it reaches its peak in the fourth decade of life. From 4 to 20 years, an average annual rate of enlargement of the WM volume is 0.77% (Giedd et al., 1999). From 40 to nearly 50 the WM volume is stable. Some authors argue that the WM volume starts to drop at the age of 50 (Pagani et al., 2008) while others found that it occurs at 60 years (Good et al., 2001). From our study, there is a shallow decrease in the WM volume after 60–65 years (refer to the curve in green in Figure 2E). A negative effect of aging on the WM volume accumulates throughout the years. The WM volume decreases by 13% between 40 and 70 (Courchesne et al., 2000). A postmortem study also supports *in vivo* findings by revealing a decrease in the WM volume by 15% in the group 62–90 years vs. 18–57 years (Tang et al., 1997). In adults, the decrease in the volume is smaller for WM than for GM (Ge et al., 2002).

A comparison of different age groups showed a large difference in the WM volume between the young (22–40 years) and middle-aged subjects (41–59 years). Moreover, in most brain regions, the WM volume was greater in the middle-aged population than in the young group. The WM volume was lower in an older generation (60–78 years) in contrast to the middle-aged people. In this research, a linear regression analysis indicated a gradual increase in the WM volume before 40 years of age with its peak at around 50, and a rapid decline after 60 years (Liu et al., 2016).

**White matter hyperintensities** are among the most common findings in the elderly brain. The severity of the lesions of WM varies widely in Older adults. It can be assessed with a volumetric method or visually on the rating Fazekas scale. A correlation of WMHs progressions with age was two times higher when studied with a volumetric method (Van den Heuvel et al., 2006). Some studies showed a strong association between the spread of WMHs and the results of electrophysiologic assessment as well as various frontal lobe functional measurements (Salat et al., 1999). We cannot justify this with the findings of the current study. The PTs we used are reflective of EF that also has frontal lobe representations. But, we did not find significant correlations of psychophysiological performance either with age or with functional performance in the tests.

There is an ongoing discussion on the association of the prevalence of lesions with aging in a healthy population. The formation of WMHs may serve as an indirect indicator of pathologic changes because the lesions usually represent ischemic insult (Salat et al., 1999). The list of factors leading to WMHs includes myelin pallor, gliosis, atrophy of the neuropil, subclinical ischemia, etc. (Raz and Rodrigue, 2006).

White matter hyperintensities are more often detected in older adults. In a study, only 20% of young examinees (21–30 years) had WMHs, whereas 100% of people from 71 to 80 years had them. Moreover, the size of lesions was also positively correlated with age (Christiansen et al., 1994). Another study discovered WMHs in 92% of patients over 60 years and in 22% of subjects aged from 0 to 20 years (Awad et al., 1986). An examination of healthy subjects concluded that WM lesions were uncommon in people below 55 years, but after 55 the appearance of lesions increased 10-fold. The total prevalence of WMHs was 5.3%. WMHs with a periventricular location were found in 3.7% of examinees, and WMHs in centrum semiovale in 3.7% cases. The least number of WMHs was found in the subjects between 16 and 25 years, and the highest prevalence of the lesions was seen in people from 56 to 65 years old (Hopkins et al., 2006). The formation of a new WMH was not rapid (De Groot et al., 2000).

When studying the lesions, radiologists assess the severity (size), they also report the location and the progression of WMHs in follow-up studies. An objective of a study was to identify the number and size of the lesions in three age groups of the elderly. All the groups had mostly small WMHs (1–3 mm). Notably, the number of the lesions increased significantly from the 6 to 7th decade, whereas it rose slightly between the 7 and 8th decade of life. On average, the examined subjects in each age subgroup had 1–2 large (>10 mm) and 2–5 medium (3–10 mm) lesions (De Groot et al., 2000).

Other researchers studied deep WMH (DWMH) and periventricular WMH (PVWMH) lesions separately. They found that PVWMH contributes to 2/3 of the total WMH. Both DWMH and PVWMH correlate significantly with a GM reduction in people over 60. The interrelation between the GM volume and the WMH load has a regional specificity, e.g., DWMH correlates with a cortical GM reduction to a greater extent than PVWMH does (Wen et al., 2006).

A study of a population of 73 ± 1 years old showed a stronger association of WMHs with global deep brain atrophy than with superficial atrophy. The volume of the lesions correlated negatively with the total brain volume and positively with the intraventricular volume. At the same time, WMHs increase, while the WM and GM volumes go down (Aribisala et al., 2013).

The discrepancies between the results of the previous studies can be explained by different approaches to the selection of the study cohorts. Supposedly, some of them might have included the clinical population with a background vascular pathology. To analyze the impact of age on the brain structure with a minimal additive influence of confounders (cardiovascular pathology, etc.), we followed the inclusion criteria listed in subsection 3.1 and studied the examinees reflective of the healthy population.

### 5.2. Sex Differences in Age-Related Changes of Brain Structure

#### 5.2.1. Skull Morphometry

Total intracranial volume and various brain regions are not equal in size between men and women. An analysis of subjects aged 16–65 years indicated that the changes in TIV were not associated with age in female participants, while men showed a trend toward a lower intracranial volume, although the correlation was not significant (Blatter et al., 1995).

The differences in the skull size account for the discrepancies in the brain parenchymal volume between sexes. For example, in a cross-sectional study of people aged from 31 to 84 years, men had a 15% larger volume of the brain. Temporal lobes and hippocampi in males were also much larger in volume (14 and 8%, respectively) (Scahill et al., 2003). Another study of a similar cohort reported a negative correlation between age and the total brain volume with identical slopes of the regression for men and women (Gur et al., 1991).

#### 5.2.2. Subarachnoid Space

Although sex differences have been described in the size, symmetry, and function of several brain structures, only a small number of imaging studies have examined the impact of sex on brain aging *in nonpatient samples* of living humans (Shaw et al., 1984; Gur et al., 1987). Some authors found an age-associated increase in lateral fissure CSF volume to be significantly greater in men than in women. From their analysis, for ages 65 to 95 years, men had an increase in the lateral fissure volume of approximately 80%, while women had an increase of only approximately 37% (Coffey et al., 1998). The lateral fissure CSF volume is a marker of frontotemporal atrophy that evidently has cognitive implications. Out of this, one may expect different functional outcomes of brain aging for females and males.

Similar data were found in another study. It indicated that correlations between age and subarachnoid CSF volume are higher in men (*r* = 0.653) than in women (*r* = 0.545), although these correlations were not statistically compared (Blatter et al., 1995). In contrast, studies that examined peripheral CSF volume found that sex does not affect the age-related increase (Sullivan et al., 1993; Murphy et al., 1996; Raz et al., 1997).

Later studies reported a trend toward a larger rate of CSF increase in women (Lemaître et al., 2005), this contradicted the hypothesis that the brains of men are more vulnerable to aging (Coffey et al., 1998). The same authors highlighted an annual rate of CSF increase for men and women of 3.3 and 4.0 *cm*^3^ per year, respectively. According to them, in women, sulcal and ventricular CSF compartments rise in volume faster.

#### 5.2.3. Brain Ventricles

Previous reports suggested that brain atrophy is associated with aging and that there are sex differences in it. A study was carried out on a group of adults from 24 to 80 years old. The authors observed a marked difference between sexes in mean ventricular volumes for the youngest age group (24–40 year). A gradually progressive increase in ventricular size was seen between the youngest and middle age (41–60 year) groups (Matsumae et al., 1996). The authors noted a higher marked percentage increase in ventricular volume of men (90%) compared to women (60%) between the middle age and oldest groups. In contrast to this analysis, others did not find any difference in linear measures of ventricles for sexes, but the cranial cavity was larger in men (Murphy et al., 1996). Other examiners illustrated a relatively higher mean of fourth ventricle anterior-posterior length in males (Ambarak et al., 2020).

In a combined study with two designs, the report suggested that the ventricular volumes and the ventricle-to-brain ratio was larger in men and in the older group. The authors did not observe an obvious effect of the hemisphere (right vs. left) for both estimates (Resnick et al., 2000). An average rate of the enlargement of the ventricular volume was 1255 *mm*^3^/*year* in the cross-sectional study vs. 1,526 *mm*^3^/*year* in the longitudinal study. Consistent with the previous report, Kaye et al. (1992) found an approximately constant 20% rise in the ventricular volume every 10 years in both sexes. The ventricular volume was correlated significantly with the performance in cognitive tests except for the verbal scale scores. A steep increase in the volume of ventricles began in the fifth decade in men and in the sixth decade in women. *This indicates that sex is an important co-founder of age-associated central cerebral atrophy*.

Whilst previous reports suggested different patterns of age-related changes for sexes, some authors highlighted a need for more empirical evidence on left ventricles. They conducted a cross-sectional study of 19–32 year-old healthy young adults and 45–58 year-old healthy middle-aged adults. A smaller left ventricular mass, wall thicknesses, volumes, and cardiac output was observed in women. Furthermore, the left ventricular volume in the middle-aged group was smaller compared to the young adults. There was no statistical difference in the left ventricle structure and its mechanics between young and middle-aged adults, but the effect of age on ventricle volume and cardiac output was statistically significant (Nio et al., 2017). These findings are in agreement with another study of healthy, non-obese, normotensive volunteers of 18–55 years. From the study, normal aging from 30 to 60 years was associated with a decrease in the left ventricular volume and length, but there was no change in the short-axis diameter, implying an increase in left ventricle sphericity (Mottram et al., 2010). A comparison of the ventricular and sulcal CSF and laterality showed that the aging effect was greater in men (Condon et al., 1988).

#### 5.2.4. Gray Matter

**Total gray matter**. Men and women have distinct trajectories of GM change. As an example, in women, a peak in GM development happens 3 years earlier (Allen et al., 2003). The total GM volume reaches its maximum at the age of 8.5 in females and 10.5 in males (Lenroot et al., 2007). The trajectories of GM volume changes are not consistent between sexes and they can be lobe specific (Tanaka et al., 2012).

A decline in the total GM volume is faster in men. However, there is no consensus on the issue whether the sex disparity is significant (Taki et al., 2004) or not (Ge et al., 2002). Females aged 8–23 years have a lower GM volume and a higher GM density than males (Gennatas et al., 2017). Healthy men aged 55–92 years have a steeper decline in the volume of the frontal, parietal, and occipital GM (Armstrong et al., 2019). The same tendency is found in a broader age range from 17 to 79 years (Good et al., 2001). In contrast to this, other studies showed that during development (Yurgelun-Todd et al., 2002; Tamnes et al., 2013; Narvacan et al., 2017) and decline (Smith et al., 2007; Tamnes et al., 2013; Narvacan et al., 2017) there is no association of the GM volume with age.

There is no agreement on the rate of GM change. The GM (2.2 *cm*^3^/year) and WM (1.7 *cm*^3^/year) volume significantly decreased with age, and the decline in the normalized GM volume was faster in women (0.20% per year) than in men (0.12%) (Lemaître et al., 2005). A longitudinal study verified a larger yearly rate of GM atrophy in women (–4.7*cm*^3^) than in men (–3.3 *cm*^3^) (Crivello et al., 2014).

A supposed explanation for the disparity in the findings is the following. GM% is markedly higher in men in the third decade and in women in the sixth decade. There are no sex differences in GM% in the fourth and seventh decades (Taki et al., 2011). Another possible reason for the discrepancies is that changes in the GM cortex and subcortical regions are not considered independently in the studies. A sex-by-age interaction is not found in the subcortical GM volume (Koolschijn and Crone, 2013) whereas the GM volume of cortical regions decreases faster in men (Koolschijn and Crone, 2013; Jäncke et al., 2015).

**Cortical gray matter**. Puberty-related maturation of cortical GM, hippocampus and amygdala is different for girls and boys. The right and left cortical GM volumes in females are much smaller in the mid- and late-puberty than at an early stage. Contrarily, in boys, puberty could not be used as a predictor of the overall cortical GM volume (Bramen et al., 2011). Mapping of cortical thickness in individuals aged 7–87 years reveals cortices of the right inferior parietal and posterior temporal regions to be thicker in women (Sowell et al., 2007).

*Parahippocampal and lingual gyri* of males reduce in size faster (Taki et al., 2011). The vulnerability of the *inferior temporal cortex* to aging is also higher in men (Raz et al., 2004). Men aged 18–49 years exhibit a faster atrophy of cortical GM especially in *dorsolateral prefrontal regions* (Gur et al., 2002).

#### 5.2.5. White Matter

**Total white matter**. Generally, women have a notably greater WM volume. Changes in the WM volume do not depend on age and sex in young and midlife adults (Liu et al., 2016). The absolute and normalized WM volume decreases at the same rate in elderly men and women. On average they lose 0.11% of the WM fraction per year (Lemaître et al., 2005).

Elderly men and women differ in the decline in the WM volume. 67-year-old men have a faster (–0.72%) loss of the WM volume than women of the same age (–0.60%). In contrast to this, 85-year-old women suffer a more rapid decline (–0.45 vs. –0.23%) (Sigurdsson et al., 2012).

**White matter hyperintensities**. The WM lesions correlate with brain atrophy more significantly in women (Wen et al., 2006). In subjects with a history of cardiac diseases a greater fraction of WMHs is observed in women compared to men (2.8 vs. 2.4% of WM). Men and women over 70 have a positive association of the WMHs volume with age (Fatemi et al., 2018). A cross-sectional study examined people between 60 and 90 years in order to identify the prevalence and severity of WMHs in both sexes. All subjects had a positive correlation between age and prevalance of the lesions. There were more subcortical and periventricular WM lesions in women (1.45 vs. 1.29 ml; mean grade 2.5 vs. 2.3). The severity of the lesions was also higher in women (De Leeuw et al., 2001).

#### 5.2.6. Features of Neurofunctional Age-Related Changes

Studying the divergence of psychophysiological markers of aging, we found that the variance of reaction time in PTs is heteroscedastic across the lifespan. Some dependent variables (CVMR_mean, DMT, AST_mean, and RMO_mean) differ insignificantly between the age groups. Other psychophysiological attributes (e.g., IRT_mean, TRVI) are heteroscedastic with regard to age. Interestingly, if the Adolescents group is removed from the observation, the majority of PT results become homoscedastic.

These facts show that the cognitive functions involved in PTs undergo age-related changes that may differ in the onset, pace, and extent across different domains. The greater variability in adolescence may be associated with the combined effect of a huge number of multidirectional factors: neurodevelopment, the pubertal hormonal changes, the impact of education, the career start and the adjustment to the society. Recent findings justify this assumption. A significant improvement in consistency of the movement tasks is observed across childhood into adulthood (Getchell, 2006). In general, heteroscedasticity of performance metrics is a known physiological outcome of senescence. A supposed reason for it is age-related diseases and a rise in complexity of human behavior through lifetime (Vaillancourt and Newell, 2002). To account for the performance heteroscedasticity, some researchers model the within-individual variance of a function as a power of age (i.e. as *age*^*x*^, *x* ∈ {1, 2, 3}) (Coltman et al., 2002).

The same factors underlie an increase in intraindividual SD of responding to the tasks, e.g., distribution indices in EFTs. Though the standard deviation and heterescedasticity are different metrics, they have a common pathophysiologic basis. Our results along with other studies replicate findings of a greater reaction time inconsistency in older adults (Vasquez et al., 2016). As seen from the scatter plots in Figures 5C,D, 6C,D the variance of reaction time in PTs changes as a U-shaped function of age with optimal performance (i.e., minimal variance) in the age range of 35–45 years. RMO test is an exception to this trend (refer to Figure 7B). The deviation of response time in the test is minimal in the age range of 25–35 years. The RMO test provides us with conflicting results that reflect the equilibrium between the sympathetic and parasympathetic reactions. In contrast, other PTs from the battery we designed describe executive functions.

### 5.3. Proper Model to Generalize Age-Related Changes

The comparative analysis of Figures 5, 6 shows that there are two patterns of age-related change in performance in psychophysiological tests. The results of PTs reflective of information processing speed have an evident “U-shaped” trend. A second-order polynomial expansion of age improves the fit of the data if compared to a first-order function. The dependent variables representing task switching and inhibitory control are shown in Figures 5E, 6E. The trend of change is linear and the fit of the data does not improve after resorting to a second-order polynomial dependence on age. To explain this, we compared the functional outcomes of aging with the volumetric changes observed in the major brain compartments.

**Linear dependence**. From the literature, the first degree equations can be used to model the regional effects of age on the CSF volume (e.g., the expansion of the Sylvian and interhemispheric fissures, and others) (Good et al., 2001). From our data, the WM volume and the proportion of WMHs to total WM change almost linearly throughout life (refer to Table 5). Psychophysiological metrics reflective of the task switching and inhibitory control (DMT, TRVI) also vary across the lifespan as the first power of age. The tendency of age-related changes is different for total GM and its cortical portion.

A tendency toward enlargement of the interhemispheric fissure is a supposed explanation for the linear slowing of decision-making across the lifespan. The enlargement may serve as a morphologic correlate of task switching and inhibitory control. A neural center of this cognitive subdomain is allocated within the medial wall of the frontal cortex, in particular, in the supplementary motor area. The evidence coming from the results of the "go/no-go" test suggests that premotor regions of the medial wall of the frontal lobe play a central role in response inhibition (Mostofsky and Simmonds, 2008). So, the atrophy of these neural centers accounts for the enlargement of the interhemispheric fissure. However, researchers draw attention to the fact that the *methods assuming linearity or even monotonicity of the compared age-functions should be used with appropriate caution* (Jernigan and Gamst, 2005).

**Second-degree equation**. Researchers analyzed how brain structures change in volume with age (Jernigan and Gamst, 2005). After presenting the volume as a quadratic function of age, one may observe effects depicted with either a U-shaped or inverted U-shape line.

*The inverted U-shaped line* is characteristic of the age-related changes of the WM bundles. For this reason, it is typical of WM and hippocampus which is composed of cellular heaps and WM fibers (Lemaire et al., 2013). Till roughly the age of 60–65 years accumulation of WM exceeds its hidden atrophic changes. The period is followed by the WM loss because of myelin breakdown and progressive degradation of the WM fibers. In prior research, myelin breakdown was found to be closely related to processing speed (Lu et al., 2013). Demyelination along with other WM changes underpin age-related slowing (Nilsson et al., 2014).

*The U-shaped line* is typical of the age-related variance in the GM volume and the structures rich in patches of neuronal cells. The current study showed tendency for overall GM and the GM cortex. Another study (Lemaire et al., 2013) justified this for the deep GM structures, e.g., thalamus, accumbens, lenticular and caudate nuclei. The structures are rich in cell bodies that cannot grow significantly in size as the neural fibers do. The loss of the neuronal cells is irreversible. For this reason, it is impossible to interpret a rise in a polynomial trendline of degree two in advanced age as atrophy of GM leads to the decay of the cells. This means that quadratic functions are not totally accurate in describing GM atrophy.

**Third-degree equation**. In (Lemaire et al., 2013) the volume of GM nuclei as a cubic function of age fits the data better than the quadratic function. After the authors applied it, no enlargement of the GM structures is seen in advanced age.

### 5.4. Associated Changes of Brain Structure and Function During Aging

Evidently, there is an association of brain atrophy with the cognitive changes. Scientists tend to establish exact structural correlates of the functional decline. Such knowledge would help radiologists to report cases. Bioengineers have a particular interest in finding the structures that are most vulnerable to change. An additional research question is the reliability of the structures as biomarkers of age-related cognitive changes. The biomarkers can reduce the complexity of AI models for automatic classification of diagnostic images (e.g., brain MRI).

There is a conceptual model of cognitive aging that explains how the combined effects of the adverse and compensatory neural processes produce varying levels of cognitive function. According to the model, the structural brain aging directly affects age-related cognitive decline. However, the rate of decline is not common across different brain tissues. The level and rate of structural brain changes correlates with cognitive abilities in later life (Naik et al., 2017; Spreng and Turner, 2019).

An association between the structural and cognitive changes in the brain differs among its cognitive domains and regions. This is why the lifelong dynamics should be discussed for each brain compartment separately.

*Gray matter*. Age-related cognitive changes correlate with the baseline volumes of GM except for the occipital volume and intracranial volume (Fletcher et al., 2018). A study conducted on adults between 21 and 76 proved an association of age-related cognitive decline with the GM volumes, especially in the anterior cortical brain regions. Smaller lateral GM volumes are associated with a poorer executive function in the participants older than 40 (Zimmerman et al., 2006).

*White matter*. An earlier study suggested that WM was not impacted in normal aging (Ziegler et al., 2010). Other studies came up with contradicting findings and demonstrated changes in the volume, integrity, and overall health of the brain. WM can be the most valuable predictor of the transition from normal aging to neurodegenerative disease (Spreng and Turner, 2019). Measures of brain structural covariance have proved to be powerful predictors of cognitive capacity in normal aging and potent biomarkers of transition from normal aging to neurodegenerative disease. According to a study, decreased brain functioning compensates for cognitive performance in older adulthood (Cabeza et al., 2002). There is evidence that better performance in neuropsychological tests is associated with a greater volume of total WM. The WM volume of frontal lobes on both sides modulates performance in memory tests. The left frontal lobe is exclusively associated with age and performance in EFTs (Brickman et al., 2006).

*White matter hyperintensities*. In a study, elderly subjects with a greater volume of WMHs had a slower processing speed. An enlargement of the summary volume of the lateral ventricles was indicative of a worsened episodic memory, processing speed, and semantic memory tasks (Dong et al., 2015).

*Brain volume*. A few studies indicated a direct association between a decrease in brain volume and cognitive decline (Sluimer et al., 2008). The decline in brain tissue volume and an increase in the CSF was associated with lower cognitive performance (Cardenas et al., 2011). A study reported an accelerated tissue loss in subjects with mild cognitive impairment compared with the normal decline (Driscoll et al., 2009). Some researchers found that gray or WM depreciation may occur in different areas of the brain causing differentiated effects on cognition (Taki et al., 2011). Though the associations may be common for the gray and WM whole-brain volumes, the functional effect may be different because of their roles in the underlying neural networks (Ziegler et al., 2010).

*Sex-related differences* are present in structure-function associations. In the age range of 18–45 years, men do not present a significant correlation of intracranial volume with verbal performance, whereas there is a correlation of cranial volume with verbal performance in women (Gur et al., 1999).

## 6. Strength and Limitations

We limited the study to the major brain compartments instead of examining all the possible regional effects of brain aging. The idea was to correlate the psychophysiological performance with metrics of the brain atrophy before making a more detailed analysis. The reason why we resorted to this approach is that brain segmentation may provide a big amount of data. Dealing with them may be challenging as there is no clear understanding which brain regions are employed in the psychophysiological tasks of our battery. Today's neuroscience lacks an empirical justification of the structural correlates of cognitive performance. To address the issue in our studies, we combined the evidence on the neurobiology of aging from MRI and PTs.

On the positive side, we performed a proper statistical analysis to infer that the effects of age differ across the brain compartments and cognitive subdomains. We took into account the compositional nature of brain volumes which are non-negative and sum to the TIV. To compare the rate of change, we used a uniform percent loss per year as an index, because the absolute volume loss was more serious in a large structure than in a small one. Other authors confirm the usefulness of ratios for characterizing compositional data, particularly, in comparing structures with volumes on different scales (Jernigan and Gamst, 2005). The PTs we applied cover distinct cognitive functions. The methodology of the current study was designed to detect change in the brain morphology and EF. The whole set of solutions enabled us to justify the findings.

## 7. Conclusion

- WM and the proportion of WMHs to total WM changes almost linearly throughout life. Total GM and its cortical portion follows different trends of age-related change. An enlargement of the interhemispheric fissure might explain the linear retardation in decision-making across the lifespan. The premotor regions of the medial wall of the frontal lobe play a central role in response inhibition. Atrophy of these neural centers accounts for the enlargement of the interhemispheric fissure.
- After presenting a brain volume as a quadratic function of age, one may observe the effects depicted with either a U-shaped or inverted U-shape line. The inverted U-shaped line is characteristic of change of the WM bundles. The U-shaped line is typical of variance of the GM volume and the structures reach in patches of neuronal cells. It is impossible to interpret a rise in the parabola in the advanced age as atrophy of GM is irreversible. For this reason, the volume of GM nuclei as the cubic function of age fits the data better than the quadratic function.
- The strongest correlation between the brain structural data and the functional outcomes is observed between total CSF% and reaction time in the IRT which is the most cognitively demanding task in our battery (*r* = 0.36). The test employs a set of cognitive domains and subdomains such as information processing, switching and inhibitory control, and attention.
- Decision-making time which reflects switching and inhibitory control is associated positively with the relative volumes of the cerebrospinal fluid and negatively with the relative volume of GM. These findings justify the reliability of the PTs that we use.

## Data Availability Statement

The datasets presented in this study can be found in online repositories. The names of the repository/repositories and accession number(s) can be found below: The datasets generated for this study are available on request at the site of Big Data Analytics Center (BIDAC) at https://bi-dac.com.

## Ethics Statement

The studies involving human participants were reviewed and approved by UAEU Human Research Ethics Committee (Notice Number: ERH_2019_4006 19_11). Approval was also obtained for the retrospective analysis of the data. Written informed consent to participate in this study was provided by the participants/patients, or by the participants' legal guardian/next of kin.

## Author Contributions

YS, TH, and ML contributed to the conceptual idea of the paper. YS and ML formulated the objectives and wrote the manuscript. TH performed the statistical analysis, prepared the figures and tables for data presentation, and illustration. DS, GS, KN-V, NZ, JA, MB, and TA contributed to the literature review and data analysis. All authors contributed to the article and approved the submitted version.

## Funding

This work was supported by Aspire grant AARE19-060 (PI: ML), UAEU StartUp grants 31M442 (PI: YS), G00003264 (PI: MB).

## Conflict of Interest

The authors declare that the research was conducted in the absence of any commercial or financial relationships that could be construed as a potential conflict of interest.

## Publisher's Note

All claims expressed in this article are solely those of the authors and do not necessarily represent those of their affiliated organizations, or those of the publisher, the editors and the reviewers. Any product that may be evaluated in this article, or claim that may be made by its manufacturer, is not guaranteed or endorsed by the publisher.

## Abbreviations

cGM: cortical gray matter
CSF: cerebro-spinal fluid
CVMR: complex visual-motor reaction
DMT: decision-making time
DTI: diffusion-based MRI tractography
DWMH: deep white matter hyperintensities
EF: executive functioning
EFT: executive functioning test
FLAIR: fluid-attenuated inversion recovery
GM: gray matter
iCSF: intraventricular CSF
iCSF%: proportion of iCSF to TIV
LOEWSS: non-linear Locally Weighted Scatterplot Smoothing
MAE: mean absolute error
MMSE: mini-mental state examination
ML: machine learning
MRI: magnetic resonance imaging
OLS: ordinary least squares
PS: psychophysiological status
PT: psychophysiological test
PVWMH: periventricular white matter hyperintensities
RMSE: root mean squared error
RMO: reaction to a moving object
SVMR: simple visual-motor reaction
TIV: total intracranial volume
T1W: T1-weighted
TRVI: time delay because of visual interference
TE: time of echo
TR: time of repetition
VBM: voxel-based brain morphometry
WM: white matter
WMH: white matter hyperintensity.
