# Supplementary material for: Brain Morphometry and Cognitive Performance in Normal Brain Aging: Age- and Sex-Related Structural and Functional Changes
Source: Front Aging Neurosci. 2022 Jan 26;13:713680. doi: 10.3389/fnagi.2021.713680 (PMC8826453; doi:10.3389/fnagi.2021.713680)
Supplement: Supplementary file 1 [file Data_Sheet_1.pdf]

SUPPLEMENTARY MATERIAL

1 BRAIN STRUCTURAL CHANGES AND NEUROFUNCTIONAL PERFORMANCE

1.1 Dynamics of skull and brain morphometry across life

The ordinary least squares regression trendlines on Figures 2-4 illustrate a distribution of the major brain compartments over age. The linear regression trendlines on Figure 3 and 4E-4H show the brain structural changes for each age group. Table 1 presents the average data for four age groups and the results of Kruskal-Wallis test. The group data with a distribution significantly different from the entire cohort are marked with asterisks. The p-values in the right column of the table show whether there is a significant difference between all the age groups taken into analysis. **Total intracranial volume**. There is a steady decrease in the total intracranial volume from Adolescents through Young and Midlife adults to Older adults ($p = 0.0068$). A supposed reason for this is a rise in the size of the skull and body from one generation to another. The significant difference justifies a necessity to adjust the individual brain volume to TIV. This enabled us to perform a comparative study of the age groups.

Cerebrospinal fluid. Scatter plot 2A describes the lifelong dynamics of the proportion of the CSF (CSF%) to the total intracranial volume (TIV). As the remaining part of the intracranial volume is occupied by the brain, one can consider the CSF% as *a summary indicator of brain atrophy*. In general, there is a slight increase in the percentage of the CSF across the lifespan.

From Table 2, the slopes for age-related dynamics of the CSF% are significant in Young adults and Older adults (p=0.00405 and 1.04e-08). As seen in Figure 3A, the slopes for both Young and Older adults are steeper than in the other groups. This justifies that the reduction of the brain volume is most prominent during the age periods from 20 to 40 years and after 60. In contrast to this, the brain volume remains relatively stable in the life intervals before 20 years of age and between 40 and 60.

Intraventricular volume. Scatter plot 2B illustrates the rate of the intraventricular CSF to TIV (iCSF%) in conjunction with age. The rise in the iCSF% is also indicative of the atrophy of the brain parenchyma, particularly, of the white and deep gray matter. The volume of the brain ventricles increases slightly across the lifespan. The dependency between the iCSF% and age is almost linear from birth till the age of 60 years. The dynamics of the iCSF% in these years is not significant ($p > 0.05$). As presented in Figure 3B, the effect of age on the iCSF volume is prominent after 60 years, i.e., in Older adults ($p = 0.000758$).

From Figures 4A and 4E, the iCSF/CSF ratio rises up slightly with age. The ratio remains almost unchanged in all ages except for the group of Older adults. In that group an increase in the iCSF-to-CSF proportion is higher than in the overall study cohort. The p-value of 0.069 reflects a near-significant trend toward a rise in the ventricular volume in proportion to the total space occupied by the CSF in the elderly.

The bottom part of Table 2 provides a comparison of the slopes for the relative iCSF% and CSF% plotted against age. In the groups of Young and Older adults, the age-related dynamics of the iCSF% is significantly different from the one for the overall CSF% ($p = 0.00879$ and $1.57e - 07$ correspondently). There is a near-significant difference between the changes of the same indicators in Adolescents ($p = 0.069$). In all ages the pace of enlargement of the intracranial subarachnoid space is higher if compared to that of the ventricular volume. Visually the slopes for age-related changes of both the CSF% (Figure 3A) and iCSF% (Figure 3B) are steeper in Older adults than in the general population.

Gray matter. Diagram 2C illustrates the lifelong changes in the total gray matter volume in relation to the total intracranial volume (GM%). A decline in GM% is observed throughout life. As presented in Figure 3D and Table 2, a reduction of the GM% is significant in Adolescents ($p = 4.78e - 05$) and Older

adults ($p = 0.0003165$). The age range from 20 to 60 is a period of relative stability in the GM% when the percentage of GM reduces slowly ($p > 0.05$).

The proportion of the cortical gray matter to the skull volume (cGM%) also reduces with age. The vacated space between sulci is filled with cerebrospinal fluid and its proportion (CSF%) increases placidly throughout life. We inverted the slopes for both the GM% and cGM% to compare them with the dynamics of the CSF% (see data for "$CSF\ vs\ -GM$" and "$CSF\ vs\ -cGM$" in Table 2).

In Adolescents, a reversed slope for the GM% loss is significantly steeper than a slope for the CSF% accumulation (0.552 ± 0.122 vs 0.135 ± 0.067; $p = 0.00346$). Presumably, there is an active accumulation of the WM volume at the time of active neurodevelopment and myelination. For this reason, the loss of the gray matter volume is partially compensated by the expansion of the white matter. In contrast to this, Older adults have a reversed slope for the GM% loss significantly shallower than the slope for the CSF% accumulation (0.246 ± 0.064 vs 0.438 ± 0.065; $p = 0.0372$). This can be explained by a significantly slower accumulation of the WM at advanced age compared to the beginning of life.

Cortical gray matter. Scatter plot 2D presents the age-related changes in the volume of the cortical gray matter in percentage to the volume of the skull (cGM%). The index continuously falls with age. In analogy to GM%, life-related changes of the cGM% are pronounced in the age below 20 ($p = 3.82e - 06$) and after 60 years ($p = 0.00302$) (see Figure 3D).

From the comparison of the slopes, the age-related loss of the cortical gray matter is significantly steeper than the pace of expanding of the subarachnoid space in Adolescents (0.512 ± 0.096 vs 0.135 ± 0.067 ; $p = 0.00182$). In opposite to this, the rate of the decrease in cGM was significantly lower than the dynamics of the accumulation of CSF in Older adults (0.179 ± 0.058 vs 0.438 ± 0.065; $p = 0.00354$). The supposed explanation is related to the rate of the white matter accumulation which is fast in minors and reduces with advancing age.

The changes in the cortical-to-total gray matter volume (cGM/GM) are also characteristic of the brain atrophy. In general, there is a trend towards a reduction of this parameter (see Figure 4B). A slump of the cGM/GM is significant in the Adolescents' group ($p = 0.000276$) as seen in Figure 4F. After the age of 20 years, the index of the cGM-to-GM remains stable with a slight linear pattern of changes till the end of life.

From the visual comparison of Figure 3C with 3D, the lifelong dynamics of the fraction of GM is almost similar to the correspondent data for the gray matter cortex. A reduction of GM% and cGM% happens at a relatively equal pace in any age group. Therefore, the slopes for these parameters do not have a notable difference ($p > 0.05$). This tendency shows a direct association of shrinking of cGM with total GM. Out of this, the age-related changes of the deep GM are supposed to be non-considerable in normal aging.

White matter. The portion of the total white matter (WM%) follows an age-related pattern of changes opposite to the one for the GM%. It rises across the lifespan but the rate of changes is not common for the age groups (see Figure 2E). The expansion of the WM is significant in Adolescents ($p = 0.00115$). This is followed by a non-significant increase in the WM% from 20 years till the end of life (see Figure 3E). However, the polynomial model of the distribution of the WM volume over age evidences a shallow decrease in the volume after the age of 60-65 years (see the curve in green at Figure 2E).

The white matter rises significantly steeper than the subarachnoid space expands in Adolescents (0.448 ± 0.128 vs 0.135 ± 0.067; $p = 0.033$). The tendency reverses in Older adults. At this age the dynamics for the fraction of the WM is significantly slower than the one for the CSF (0.013 ± 0.03 vs 0.438 ± 0.065; $p = 2.2e - 16$). As mentioned before, the supposed explanation is the pace of the white matter myelination

which reduces slowly across the lifespan. Due to this the slope for the CSF% ascents at a faster rate than the one for the WM% at advanced age.

To compare the dynamics of the loss of the GM and the accumulation of the WM, we inverted the slopes for the WM% (see data for $"GM\ vs\ -WM"$ in Table 2). In Adolescents the GM% slope is significantly steeper compared to the reversed slope that describes changes in the WM% (-0.552 ± 0.122 vs -0.448 ± 0.128; $p = 2.2e - 16$). The rise in the WM fraction along with the increase of the skull volume reduces the percentage of the total gray matter at the time of a rapid growth and neurodevelopment. The same significant difference is present in Older adults (-0.246 ± 0.064 vs -0.013 ± 0.03; $p = 0.565$). The dramatic atrophy of the cerebral GM in this age group may explain these changes

White matter hyperintensities. Diagram 2F shows a slight decrease in the proportion of the volume of white matter hyperintensities to TIV (WMH%) throughout life. As it is seen in Figure 3F, there is no significant rise in WMH% across the lifespan. The slopes for WMH% are non-significant ($p > 0.05$) in all age groups (see Table 2). The percentage of the white matter occupied by lesions (WMH%/WM%) remains also almost stable in all ages (see Figure 4D). We observed a significant ($p = 0.019$) decrease in the WMH%-to-WM% ratio only in the group with the age range from 4 to 20 years (see Figure 4H and Table 2). An active growth of the WM% within this period accounts for this tendency. This can be explained also by an active myelination of the white matter at the time of rapid development and maturation. The appearance of white matter lesions is a sign of demyelination which is uncommon at the beginning of life. This is quite evident from the comparison of the slopes for WMH% vs WM% in Adolescents (0.011 ± 0.012 vs 0.448 ± 0.128; $p = 0.000615$). In the following years of life, the fraction of WM foci with abnormally high intensity on FLAIR increases non-significantly.

The slopes for the CSF accumulation are significantly steeper compared to the ones for WMH in all ages except for Middle-aged adults. The accumulation of the CSF continues throughout life and has its peak in Older adults whereas the fraction of WM hyperintensities remains almost unchanged in normal brain aging. From our data, vascular lesion of the WM is not the major determinant of brain structural changes throughout life. The accumulation of white matter lesions may happen for many reasons including brain ischemia due to cerebrovascular pathology. Supposedly, such an accumulation may be viewed as a sign of a brain disease rather than a common outcome of brain aging.

1.2 Psychophysiological performance during aging

The distribution of psychophysiological tests results over age is illustrated with ordinary least squares regression trendlines on Figures 5-7.

Diagrams 5A-5D and 6A-6D show age-related changes of the dependent variables that reflect information processing speed in PTs with diverse task paradigms. The parabolic trendline colored in green shows the distribution of the test results over age better than the linear trendline drawn in red.

Figures 5E and 6E present the distribution of the derivative variables that reflect the time spent on task switching and inhibitory control (i.e., inhibiting an automotic response, making a decision, selecting the correct way to respond, etc.). The data on these scatterplots have almost linear distribution that is close to the red linear trendline.

Scatter plot 7A reports the linear distribution of the results in RMO test over the years of life. On average, the mean time estimate in this test is close to zero, and the result remains unchanged throughout life. The supposed reason for this is that RMO_mean reflects a balance between excitation and inhibition in the central nervous system. But the accuracy in the RMO test changes with age. For this reason the

variance of reaction time (RMO_variance) follows the green parabolic trendline on Figure-7B with the best performance in the age range from 35 to 40 years.

As Shapiro-Wilk test for normality with $p < 0.05$ revealed that the data were not normally distributed, we assessed the differences in the groups with non-parametric tests. Kruskal-Wallis test shows that only TRVI group samples originate from a common distribution ($p > 0.05$). In the data from other PTs, at least one group stochastically dominates the other samples ($p < 0.05$). We also assessed the homogeneity of variance for each attribute with a nonparametric Levene test for equal variances. For each age group, we tested the homoscedasticity of the data with the granulation equals to 20 years. According to the test results, CVMR_mean, DMT, AST_mean, and RMO_mean are homoscedastic ($p > 0.05$) while the rest attributes have significant differences in variance ($p < 0.05$) between the age groups.

The linear regression trendlines on Figure 7C and 8 indicate changes in psychophysiological performance for each age group. From the diagrams it is seen that the results in psychophysiological tests follow a common age-related tendency. The tendency is characterized with a considerable improvement in the performance in the tests from 4 to 20 years of age. In adolescents the slopes of all these variables are negative and steep (p-value ranges from $2.6e-07$ to 0.0475; see Table 2). This is because the speed of the reaction increases considerably and the reaction time drops at the time of neurodevelopment.

The performance starts worsening after 20 years of age. *In Young adults* the slopes are positive and quite steep (p-value ranges from $2.96e-07$ to 0.0012). *In Midlife adults* and *in Older age* there is a slight increase in the reaction time in PTs. The slope for IRT_mean which is significant in Older adults ($p = 0.035$) is an exception.

It is remarkable that the more cognitively demanding the task is, the more pronounced the association of the dependent variable with age gets. This is evident from the comparison of reaction time in SVMR, CVMR and AST test. Reaction time in IRT test has the most well-defined trend of age-related changes. The results of this test are most evident of the age-related decline.

TRVI varies slightly across the lifespan. The general lifelong trend in its changes is common to all PTs with a slight decrease in Adolescents and a moderate rise in the other age groups. The slopes for TRVI are non-significant. The p-value is near to significant ($p = 0.055$) only in Older adults.

Reaction time in RMO test is not affected by age after 20 years. A suggested explanation to this comes from a completely different paradigm of testing RMO compared to the other tasks. During RMO test the examinee is asked to respond to events that happen at some expected time moment. Contrary to this, before taking other tests the individual is instructed to wait for an unexpected event to come (e.g., an appearance of a targeted stimulus in ASR and IRT or a light flash in SVMR and CVMR tests).

1.3 Sex differences in lifelong dynamics of brain morphometry

The data in Table 3 justify the equal distribution of female and male study participants over age. This enables us to compare their brain morphometry data for them.

Table 1 contains the data for both sexes. In the table every age group has p-values that reflect how significantly females and males differ. For absolute volumes of brain compartments (e.g., GM, WM, CSF, etc.) Mann-Whitney U-test has provided a significant difference ($p < 0.05$) between sexes. There is much evidence that the skull size accounts for the major part of these findings as the difference between sexes in TIV was most prominent ($p \leq 0.002$) if compared to the brain parts.

After being adjusted to the skull volume, the relative volumes of the brain compartments (e.g., GM%, WM%, CSF%, etc.) do not differ significantly for both sexes in Adolescents and Young adults. The

tendency changes after 40 years of age. The percentage of the total GM is significantly higher in women than in men in middle-aged individuals (31.85 ± 3.96 vs 29.46 ± 2.86; $p = 0.009$) and in old adults (30.94 ± 3.65 vs 28.87 ± 2.38; $p = 0.0145$). The data for the cortical GM differ significantly between sexes in Midlife adults (21.64 ± 3.68 vs 19.54 ± 2.91; $p = 0.0142$). This can be explained by the sex difference either in the speed or in the onset of the atrophic changes in the gray matter. Atrophy of the gray matter either starts earlier or goes faster in males and it accounts for a significant difference in GM-to-WM ratio between sexes in Midlife adults (77.43 ± 15.81 vs 70.17 ± 14.65; $p = 0.0375$) The tendency is near to significant in Older adults (76.18 ± 17.77 vs 68.22 ± 12.14; $p = 0.0741$)

A marked sex difference in the proportion of the total CSF (22.68 ± 3.33 vs 26.79 ± 4.94; $p = 0.0036$) and iCSF(1.75 ± 0.79 vs 2.54 ± 1.02; $p = 0.0033$) justifies that elderly men are much more prone to age related brain atrophy than women of the same age. There is a significant difference in the iCSF-to-CSF ratio between sexes after the age of 60 years (7.59 ± 2.72 vs 9.25 ± 2.7; $p = 0.0076$) The distribution of WMH-to-WM index has its sex-related features that are near to significant in the age range from 40 to 60 years (2.76 ± 1.37 vs 3.22 ± 1.19; $p = 0.0595$) and after the age of 60 (2.58 ± 1.51 vs 3.04 ± 1.1; $p = 0.0511$).

1.4 Mathematical models of the age-related changes

As mentioned before, the ridge regression model applied to the linear and non-linear function of age (see Equations 9-10) allowed us to build two types of approximation functions for various attributes. These are a straight line and a parabola (second-order line). We supplied scatter plots in Figure 2 and Figures 4-7 with trendlines both for linear and for polynomial kernel ML non-linear models and their 95% confidence intervals. This helps us to select the best model.

From the diagrams for voxel-based morphometry data in Figure 2 and 4A-4D, the data are more scattered across the lifespan than the psychophysiological variables. For this reason it is hard to select a proper mathematical model by basing the life-long changes on a visual trajectory.

From the visual appearance of the scatter plots for psychophysiological tests (Figures 5-7) it is quite evident that the data can be grouped into two categories. *The first category* contains variables that describe age-related changes in reaction time in SVMR, CVMR, AST and IRT tests and the variability of the time in all the tests including RMO. This category has a U-shaped form of distribution over age and polynomial kernel regression model describes it in a better way. The variables that reflect either task switching (DMT, TRVI) or the balance of processes in the central nervous system (RMO_mean) constitute *the second category*. Linear models reflect the changes of these variables throughout life reliably.

1.4.1 The performance of the linear and non-linear models

To justify the selection of preferable mathematical models, we compared the potential of the models to predict brain structural and functional changes throughout life. For this we calculated performance metrics (see Table 4). Because of the statistically significant ($p < 0.05$) association of the aforementioned variables with age, the age of the examinee can be used as a predictor in the regression models.

The low R-squared (R^2) values of the prediction models indicate a high variability around the regression line. This can be explained by the nature of our data: the psychophysiological performance is unstable and it reflects the adjustment of an individual to the living conditions. Nonetheless, the reproducibility of the PTs and their informative value allow us to consider the tests as a screening tool for psychological disadjustment and cognitive decline (1).

The attributes with the clearly observed quadratic trendlines ($SVMR\_mean$, $CVMR\_mean$, AST_mean, IRT_mean) show some increase in the accuracy of the prediction obtained with the second-degree polynomial function of age. The bigger the curvature of the parabola is, the larger the dissemblance between the performance of the linear and non-linear model will be. The performance metric that we use to compare different models is the proportion of mean absolute error to the range of the values studied ($MAE/range$). One may rank the models with regard to the distance between the performance of the models (see Table 5).

In the left column of Table 5, we put a list of psychophysiological variables ranked according to the distance between the performance of linear and non-linear models: $SVMR\_mean$, AST_mean, IRT_mean, $CVMR\_mean$, $TRVI$, DMT, RMO_mean. The variables at the top of the list reflect *information processing speed*; they are used for cognitive processing assessments. The life-long changes in the tests match polynomial trendlines in a better way than the linear ones. In contrast to this, the variables at the bottom of the list (DMT, $TRVI$) reflect the performance of another cognitive subdomain which is *task switching and inhibitory control*. Their age-related distribution almost fits the linear model. Both the first and second-degree polynomial function models for RMO_mean have an equal performance metric because the results of RMO test do not depend on age.

In the left column of Table 5 we ranked the morphological variables with regard to the distance between the performance of the models: cGM/GM, CSF%, GM%, iCSF/CSF, WMH/WM, WM%. In general, age-related changes of the total GM ($GM\%$) and its cortical part (cGM/GM) follow the quadratic trend of retardation. The same is true for the total CSF ($CSF\%$) and the portion of its intraventricular part ($iCSF/CSF$). Linear models can describe the over-age distribution of the total WM ($WM\%$) and its lesions (WMH/WM) because a quadratic equation does not provide an advanced performance.

1.5 Comparison of brain structural changes with dynamics of psychophysiological performance

1.5.1 Association between brain volumetric data and functional outcomes throughout life

Figure 9 shows coefficients of correlation between the brain volumetric data and the major dependent variables of the battery of PTs used. From this diagram, there is a strong positive association between the total volume of the CSF and the latency of reacting to the stimuli presented in SVMR, CVMR, IRT, AST tests. A positive dependency is also observed for the volume of brain ventricles and reaction time in the tests. As seen from the diagram, both the CSF% and the iCSF% are positively correlated with age (r-values are 0.8 and 0.56 correspondently). This justifies the indexes as markers of age-related brain atrophy. As the correlation between the CSF% and age is the strongest one (maximal r value), the latter can be considered as the most sensitive marker of atrophy.

Interestingly, the strongest correlation between the brain structural data and the functional outcomes is observed between the total CSF% and reaction time in IRT test which is most cognitively demanding in our battery ($r = 0.36$). The test employs a set of cognitive domains and subdomains such as information processing, switching and inhibitory control, attention.

It is notable that the variance of reaction time in SVMR test (SVMR_variance) is not associated with age: it demonstrates a slight negative association with the relative volume of WM (WM%; $r = -0.11$). There is also a moderate positive association between SVMR_variance and the relative volume of the total gray matter (GM%; $r = 0.21$), particularly its cortical part (cGM%; $r = 0.23$).

SVMR test is the easiest test in our battery as it utilizes only information processing with a minimal load on other cognitive functions. The mean reaction time in this test (SVMR_mean) has a moderate positive

association with the relative volumes of the cerebrospinal fluid (for CSF% $r = 0.14$; for iCSF% $r = 0.18$) and the gray matter (for GM% $r = 0.16$; for cGM% $r = 0.19$). Also it has a moderate negative statistical relationship with the proportion of the total white matter (WM%; $r = -0.14$). A better brain connectivity in individuals with a high proportional volume of the total WM may account for the faster reaction. The association is prominent in SVMR test because it does not require making a decision as in "go/no-go" test, and it does not employ attention as AST and IRT tests do. For this reason the association with the brain connectivity is straight-forward in this test.

DMT which reflects switching and inhibitory control is associated positively with the relative volumes of the cerebrospinal fluid (for CSF% $r = 0.16$; for iCSF% $r = 0.14$) and negatively with the relative volumes of the gray matter ($r = 0.13$ for both GM% and cGM%). These facts justify the reliability of the PTs used because decision-making is definitely performed in the gray matter cortex. Despite the validity of the tests, the weak associations ($r \leq 0.16$) limit the use of DMT as a biomarker of brain atrophy changes.

There is no association between the percentage of WM lesions (WMH%) and psychophysiological performance as well as no correlation is seen between WMH% and age. The mean reaction time in RMO test (RMO_mean) is associated neither with age nor with the volume of the major brain compartments.

1.5.2 Comparison of slopes for brain structural and functional changes in age groups

Table 6 provides a comparison of the slopes for psychophysiological performance with those for the structural brain changes. One may see that in all age periods except for the midlife the dynamics of the tests results significantly differ from those of the brain volumetry data. The number of significant differences between the slopes for the structural and functional estimates is the highest in Adolescents, moderate in Young adults and the lowest in Older adults. The supposed explanations to this is the following. Massive neurodevelopmental changes account for the disproportional changes in the brain structure and cognitive functioning in Adolescents. A continuous accumulation of skills and educational experience justifies the same tendency in Young adults. Due to the ongoing process of acquiring new knowledge till roughly the age of over 40 years, the cognitive performance of individuals may still improve at the time when irreversible structural changes are already taking place. Dramatic changes both in the brain compartments and in the cognitive performance lead to significant differences in the dynamics of the structural atrophy and functional decline.

REFERENCES

1 .E. Statsenko, I. Charykova, Psycho-physiological criteria for overtraining in athletes, Voprosy kurortologii, fizioterapii, i lechebnoi fizicheskoi kultury (2010) 50–54.
